# Insect Gut Isolate Pseudomonas sp. Strain Nvir Degrades the Toxic Plant Metabolite Nitropropionic Acid

**DOI:** 10.1128/aem.00719-22

**Published:** 2022-09-26

**Authors:** Magda A. Rogowska-van der Molen, Dmitrii Nagornîi, Silvia Coolen, Rob M. de Graaf, Tom Berben, Theo van Alen, Mathilde A. C. H. Janssen, Floris P. J. T. Rutjes, Robert S. Jansen, Cornelia U. Welte

**Affiliations:** a Department of Microbiology, Radboud Institute for Biological and Environmental Sciences, Radboud University, Nijmegen, The Netherlands; b Department of Synthetic Organic Chemistry, Institute for Molecules and Materials, Radboud University, Nijmegen, The Netherlands; Washington University in St. Louis

**Keywords:** nitronate monooxygenase, *Nezara viridula*, nitropropionic acid degradation

## Abstract

Nitropropionic acid (NPA) is a widely distributed naturally occurring nitroaliphatic toxin produced by leguminous plants and fungi. The Southern green shield bug feeds on leguminous plants and shows no symptoms of intoxication. Likewise, its gut-associated microorganisms are subjected to high levels of this toxic compound. In this study, we isolated a bacterium from this insect's gut system, classified as Pseudomonas sp. strain Nvir, that was highly resistant to NPA and was fully degrading it to inorganic nitrogen compounds and carbon dioxide. In order to understand the metabolic fate of NPA, we traced the fate of all atoms of the NPA molecule using isotope tracing experiments with [^15^N]NPA and [1-^13^C]NPA, in addition to experiments with uniformly ^13^C-labeled biomass that was used to follow the incorporation of ^12^C atoms from [U-^12^C]NPA into tricarboxylic acid cycle intermediates. With the help of genomics and transcriptomics, we uncovered the isolate’s NPA degradation pathway, which involves a putative propionate-3-nitronate monooxygenase responsible for the first step of NPA degradation. The discovered protein shares only 32% sequence identity with previously described propionate-3-nitronate monooxygenases. Finally, we advocate that NPA-degrading bacteria might find application in biotechnology, and their unique enzymes might be used in biosynthesis, bioremediation, and in dealing with postharvest NPA contamination in economically important products.

**IMPORTANCE** Plants have evolved sophisticated chemical defense mechanisms, such as the production of plant toxins in order to deter herbivores. One example of such a plant toxin is nitropropionic acid (NPA), which is produced by leguminous plants and also by certain fungi. In this project, we have isolated a bacterium from the intestinal tract of a pest insect, the Southern green shield bug, that is able to degrade NPA. Through a multiomics approach, we identified the respective metabolic pathway and determined the metabolic fate of all atoms of the NPA molecule. In addition, we provide a new genetic marker that can be used for genome mining toward NPA degradation. The discovery of degradation pathways of plant toxins by environmental bacteria opens new possibilities for pretreatment of contaminated food and feed sources and characterization of understudied enzymes allows their broad application in biotechnology.

## INTRODUCTION

Approximately 450 representatives within the leguminous plant family (Fabaceae) contain toxic nitroglycosides that can be hydrolyzed to 3-nitropropanol (NPOH) and 3-nitropropionic acid (NPA) acting as defense compounds which were found to be exceedingly poisonous to humans and other animals (1). Intoxication by either compound of eukaryotes causes irreversible inhibition of the essential mitochondrial enzyme succinate dehydrogenase in the tricarboxylic acid (TCA) cycle impairing the functioning of the electron transport chain and eventually leading to cell death. Likewise, NPA intoxication leads to the accumulation of toxic nitrite ions which disrupts hemoglobin’s oxygen carrying capacity (2–4).

In addition to leguminous plants, NPA is produced by many fungi within the *Penicillium* and Aspergillus genera (5). In recent years, fungal contamination with nitroglycosides due to improper postharvest storage and food preparation caused poisonings all over the world leading to serious injuries and even death (6).

A number of bacteria were reported to degrade toxic nitroglycosides by harboring nitronate monooxygenase (NMO) encoded in their genomes (7, 8). The NMO enzyme converts the conjugate base of NPA to 3-oxopropanoate (3OP) and further to the corresponding semialdehyde, releasing nitrite and hydrogen peroxide. Subsequently, 3OP can be converted by a 3OP dehydrogenase to acetyl coenzyme A (acetyl-CoA), resulting in CO_2_ generation (9). Released degradation products such as nitrite are later converted into ammonium, while acetyl-CoA serves as a substrate in the TCA cycle, thereby providing a nitrogen and carbon source for bacteria, respectively (8).

In this study, we investigated the metabolism of an NPA-degrading Pseudomonas sp. strain, Nvir, isolated from the gut of a pest insect, the Southern green shield bug Nezara viridula (Hemiptera: Pentatomidae). With the use of genomics, transcriptomics, and metabolomics, we reveal the complete NPA degradation pathway, including the metabolic fate of all carbon and nitrogen atoms in the NPA molecule. We discovered that a previously uncharacterized flavin-dependent monooxygenase gene, *pnm*R, likely encodes a nitronate monooxygenase with only 32% sequence identity on the amino acid level to previously described nitronate monooxygenases, involved in the first step of the degradation pathway. We advocate that Pseudomonas sp. Nvir isolated here and its unique uncharacterized yet nitronate monooxygenase possess the variety of biotechnological application in agriculture and the food industry as well as in organic synthesis and bioremediation (10).

## RESULTS

### Isolation and taxonomy of NPA-degrading Pseudomonas sp. Nvir.

To isolate NPA-degrading bacteria, gut systems of the Southern green shield bug *N. viridula* (Hemiptera: Pentatomidae) were dissected and pooled. To enrich NPA-degrading bacteria, the gut suspension was inoculated in selective M9 mineral salt medium containing NPA as the sole carbon and energy source. In this attempt, several isolates were obtained and further investigated. Selected strains were subsequently grown in the same medium, where NPA served as the sole carbon, nitrogen, and energy source. With this approach, only one isolate was further examined, since unlike others it was able to grow exclusively on NPA. We investigated the influence of NPA concentration on the isolate’s resistance and observed that it withstood up to 10 mM NPA (data not shown). The full genome of the NPA-degrading isolate was sequenced and had a size of 5,716,865 bp with a G+C content of 62.3%. A total of 5,464 putative genes were annotated, with 5,320 protein-coding genes (coding DNA sequence [CDS]), 70 tRNAs, four rRNA operons, and one transfer-messenger RNA (tmRNA) (see Table S1 in the supplemental material). Taxonomic analysis with the CheckM tool showed that the isolate was classified as Pseudomonas sp., designated here as Nvir (for Nezara viridula) (11). The comparison of average amino acid identity (AAI) and average nucleotide identity (ANI) of 10 Pseudomonas sp. genomes revealed that Pseudomonas sp. Nvir shared a high similarity (AAI of 94% and ANI of 88%) to Pseudomonas putida and Pseudomonas monteilii (Fig. S1 and S2).

### Pseudomonas sp. Nvir growth is supported by NPA.

**FIG 1:**
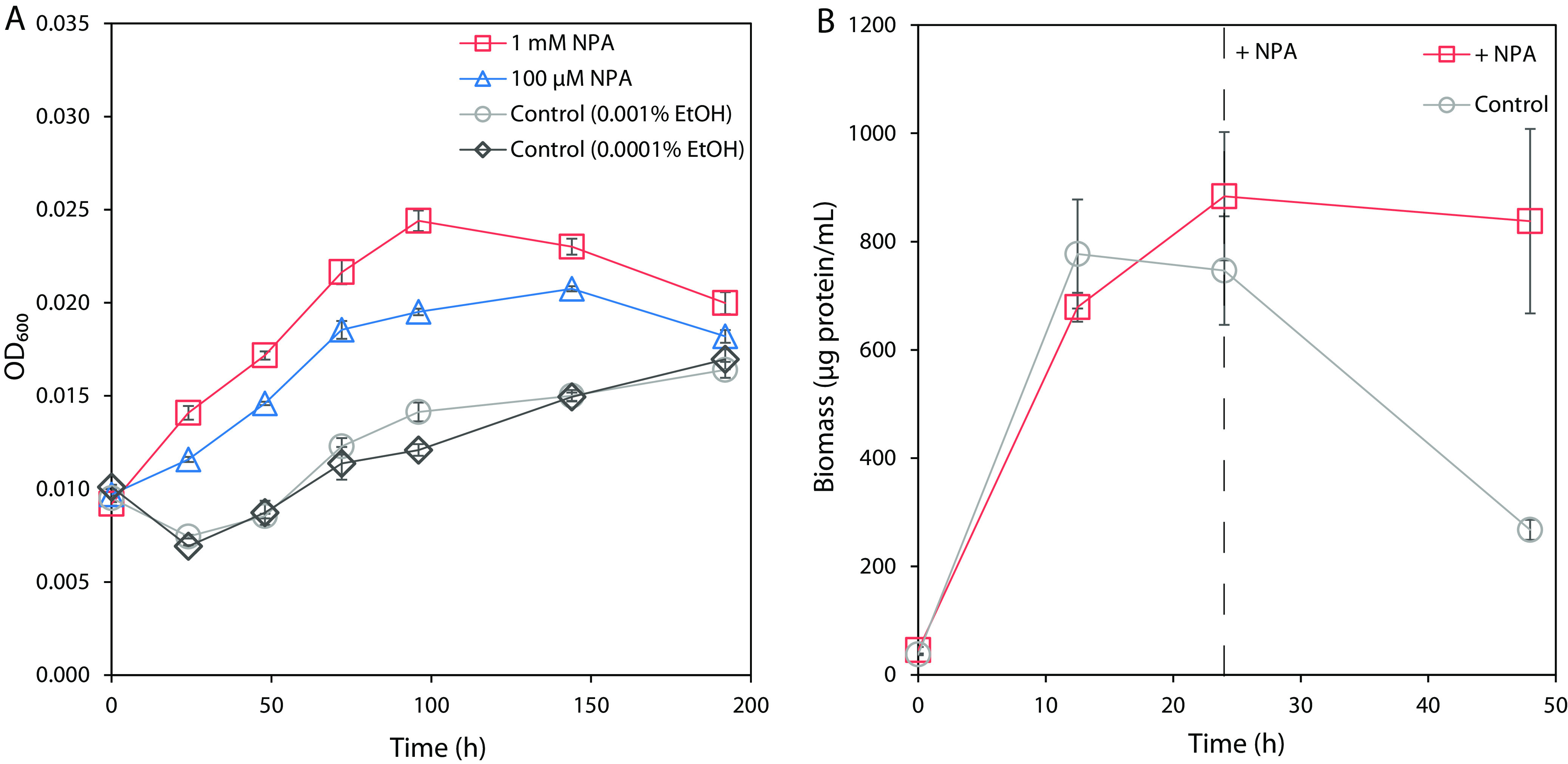
(A) Growth of Pseudomonas sp. Nvir on NPA as the sole carbon, nitrogen, and energy source. The medium was inoculated with biomass at an OD_600_ of 0.01. The growth was monitored in control cultures and in cultures supplemented with 1 mM NPA or 100 μM NPA by measuring the OD_600_ for 196 h. As controls, 0.001% and 0.0001% ethanol solutions were used, since the initial stock of NPA was dissolved in 99% ethanol. Data are presented as mean ± standard error (*n* = 3). (B) Cell pellet biomass quantification of Pseudomonas sp. Nvir during presence and absence of NPA. The medium was inoculated with ~20 μg protein/mL biomass and pregrown in an M9 mineral medium containing 20 mM glucose and 9.35 mM ammonium chloride. Subsequently, Pseudomonas sp. Nvir cultures were grown for 24 h upon both glucose and ammonia were depleted from the medium. After 24 h, 100 μM NPA was added to one culture (dashed line), designated + NPA, and compared with a control culture, where no NPA was added. The growth of Pseudomonas sp. Nvir cultures were monitored over 48 h by measuring cell pellet biomass protein content using a colorimetric assay. Data are represented as mean ± standard error (*n* = 3).

We sought to elucidate the NPA degradation pathway in Pseudomonas sp. Nvir by exploring the growth and capability to use NPA as a carbon, nitrogen, and energy source. During the isolation procedure, the strain was grown exclusively on NPA as the carbon, nitrogen, and energy source, suggesting its ability to mineralize NPA. A growth curve of Pseudomonas sp. Nvir with NPA as the sole carbon, nitrogen, and energy source is shown in Fig. 1A. We found that the growth of the isolate was not proportional to the increasing NPA concentration. In fact, instead of a 10-fold increase in growth, only roughly a 40% increase in biomass growth on 1 mM NPA in comparison with 100 μM was observed. The disproportional growth of Pseudomonas sp. Nvir might be the explained by the inhibitory influence of nitrite buildup in the beginning stage of NPA degradation as well as the toxic effect of the high concentration of NPA. On the other hand, in control cultures 0.001 and 0.0001% ethanol solutions were used, as the original stock of NPA used for these experiments was prepared in ethanol. The growth of control cultures was minimal and no difference was seen between them, indicating that the growth of biomass with supplemented NPA depended entirely on the presence of NPA in the medium.

Due to the slow growth and low biomass yield of this isolate on NPA, which hampered downstream analyses, we aimed to obtain high-cell-density cultures in further experiments. For this reason, we simultaneously grew two cultures in M9 mineral salt medium containing glucose and ammonium as sole carbon and nitrogen substrates. With this, we aimed to investigate the influence of NPA on the culture viability. After a 24-h C/N depletion step (Fig. S3), we quantified the protein content of the cell pellet biomass in the absence or presence of NPA (Fig. 1B). The supplementation with 100 μM NPA at the 24-h time point showed that the control culture decreased in biomass rapidly after 24 h, whereas the addition of NPA resulted in a steady state of biomass. The sudden decrease in the protein quantity might be caused by the cell lysis under nitrogen and carbon depleting conditions. In this experiment, only the pelleted cells were tested for the protein quantity resulting in declining protein quantity upon biomass lysis caused by the substrate depletion. The slight decrease in NPA-supplemented culture might reflect that NPA supported the culture viability, yet was not sufficient to provide growth. Moreover, the lysis likely was caused by the depletion of the growth substrates since, no pH decrease that could cause protein precipitation was seen (data not shown). These data thereby imply that NPA was used for growth under these conditions, enabling further experiments with higher biomass requirements.

### Transcriptome analysis.

**FIG 2:**
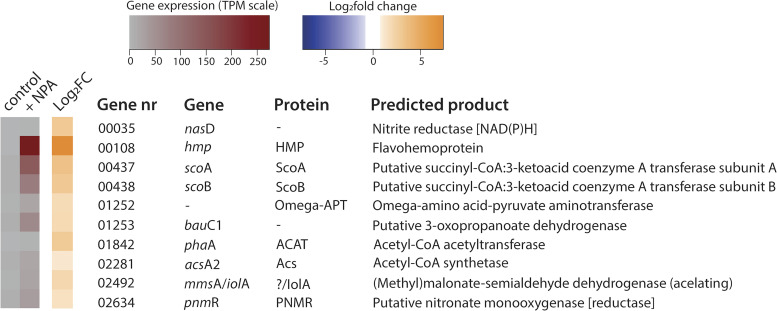
Pseudomonas sp. Nvir differential gene expression profile under NPA conditions. Pseudomonas sp. Nvir cultures grew in an M9 mineral medium supplemented with glucose and ammonium. After a 24-h C/N depletion phase, 100 μM NPA was added to the culture. Samples for the RNA extraction were collected at 1.25 h after NPA supplementation. Likewise, control samples where no NPA was added were collected at the same time point. A heat map shows the transcript expression level (TPM) in the presence (+ NPA) or absence of NPA (control) and log_2_ fold changes (Log_2_FC) in the gene expression in the 10 differentially expressed genes in Pseudomonas sp. Nvir. The complete transcriptional profile is provided in Fig. S4.

To explore the metabolic potential of Pseudomonas sp. Nvir in degrading NPA, we performed transcriptome analyses and compared differential gene expression profiles between NPA-free (control) and NPA-supplemented cultures. Both cultures were grown similarly as described in the growth experiment, and samples for the RNA extraction were collected after 1.25 h of NPA addition, where ~50% of the added NPA had been degraded (see Fig. 4 below). Control culture was sampled during the stationary state reached after growth on glucose and ammonium. Out of 4.8, 4.7, and 4.9 million obtained reads, 3.6, 3.8, and 3.8 million were mapped in control samples, respectively, whereas, in NPA-treated cultures out of 5.7, 5.6, and 6.0 million reads, 4.2, 4.0, and 4.4 million were mapped accordingly. Transcriptomic analysis revealed that 54 genes were significantly (log_2_ fold change cutoff of 1) up- or downregulated when the culture was exposed to NPA (Fig. S4); however, only 10 genes seemed to be of relevance in terms of NPA degradation (Fig. 2). The preliminary analysis revealed that upon NPA stress, Pseudomonas sp. Nvir expressed genes encoding essential enzymes involved in the conversion of NPA and its intermediates. Unexpectedly, the previously described NMO gene and the putative propionate-3-nitronate monooxygenase (encoded by the *pnoA* gene) coding for the enzyme priopionate-3-nitronate (P3N) monooxygenase, which were found to be essential in the degradation of NPA, were not found in the genome or were not expressed, respectively (8). In fact, even though the *pnoA* gene was present in the genome of Pseudomonas sp. Nvir, expression levels were not detectable in the presence or absence of NPA. Comparison of the *pnoA* gene found in this study with the homologous gene of the NPA-degrading Pseudomonas sp. strain JS189 showed that those two genes shared only 32% similarity based on amino acid sequence (8). Furthermore, in NPA-degrading Pseudomonas, genes involved in the NPA degradation were part of an operon and located in proximity with a transcriptional regulator (8, 9). The *pnoA* gene of Pseudomonas sp. Nvir seemed, however, to be randomly located in the genome, which jointly with its low amino acid identity to characterized enzymes might explain the lack of *pnoA* expression when exposed to NPA. Since our Pseudomonas sp. Nvir isolate was clearly capable of NPA degradation, these results suggested the presence of a hitherto undescribed enzyme(s) for NPA degradation. Subsequent analysis of upregulated genes within the transcriptome resulted in the identification of gene 02634, which belonged to the MSMEG 0569 flavin-dependent oxidoreductase family as classified by National Centre for Biotechnology Information (https://www.ncbi.nlm.nih.gov/). Although gene 02634 and *pnoA* share only 32% amino acid sequence identity, evolutionarily they belong to the same superfamily. Therefore, we hypothesized that the enzyme encoded by gene 02634 might harbor the same catalytic function as the NMO or P3N monooxygenase. We propose to name gene 02634 found in Pseudomonas sp. Nvir as a putative nitronate monooxygenase (reductase) with the designated gene and protein abbreviations *pnm*R and PNMR, respectively.

**FIG 3:**
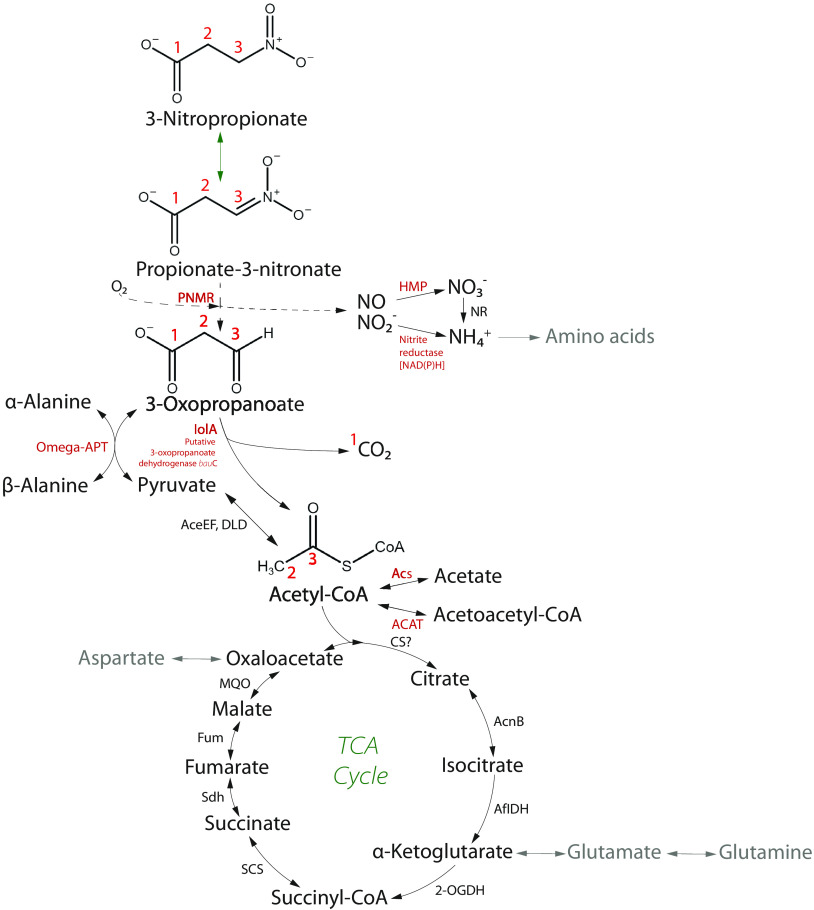
Predicted metabolic pathway of NPA degradation constructed based on the analysis of the differentially expressed genes and experimental evidence. A prediction was made on the role of the NPA in the central metabolism. Based on the experimental evidence, we showed the biotransformation of 3-nitropropionate to nitrite and nitrate, release of CO_2_, and incorporation of C2 product to TCA cycle, by quantifying aspartate, malate, glutamate, and succinate. The bioconversion pathway of intermediary products, 3-oxopropanoate and pyruvate, to α- and β-alanine and acetyl-CoA to acetate and acetoacetyl-CoA were hypothesized according the transcriptomic data set and available literature. The activity of PNMR was presumed based experimentally shown accumulation of nitrite and nitrate and its phylogenetic placement within the monooxygenase family. The complete characterization of the enzymatic activity of this enzyme remains yet to be determined. In the predicted metabolic pathway, black arrows represent reactions of NPA degradation, metabolism of generated by-products, and the TCA cycle. A green arrow indicates spontaneous biochemical conversion under physiological pH. According to the literature, gray arrows indicate amino acid biosynthetic pathways. A dashed line indicates a hypothetical pathway of the PNMR. Enzymes highlighted in red indicate encoded designated proteins highly expressed upon NPA exposure. Enzymes, in black, are identified in the genome but not highly expressed, and the “?” indicates a protein or pathway not identified in the genome. NR, nitrate reduction to ammonium probably is catalyzed by NasA and NirBD. Red numbers represent the flow of carbons based on their original position in NPA.

Furthermore, several genes encoding enzymes participating in either the direct degradation process or the transformation of the generated by-products were upregulated. According to the differentially expressed genes, a complete degradation NPA pathway was reconstructed (Fig. 3) showing the suggested fate of all atoms in the overall metabolism. Biochemically, NPA at physiological pH exists in equilibrium with its conjugate base, P3N, and both the acidic and anionic forms undergo a denitration reaction catalyzed by PNMR. NPA and P3N are oxidized to 3-oxopropanoate (3OP), losing their nitro group and releasing nitric oxide and nitrite. Nitrite is subsequently reduced by nitrite reductase to ammonium (catalyzed by the *nas*D-encoded protein), and nitric oxide is rapidly metabolized into nitrate by a flavohemoprotein (HMP). Finally, nitrate reduction (catalyzed by the *nas*A- and *nir*BD-encoded enzymes) yields ammonium, which serves as a nitrogen source for amino acids following nitrogen assimilation. Next, the intermediate product 3OP formed in the first degradation step could serve as a substrate to the α-alanine metabolism, generating β-alanine and pyruvate. Alternatively, 3OP is oxidized by (methyl)malonate-semialdehyde dehydrogenase and/or a putative 3OP dehydrogenase to acetyl-CoA and CO_2_. Acetyl-CoA is transformed into several metabolites, such as acetate and acetoacetyl-CoA, or enters the tricarboxylic acid (TCA) cycle, where it is further used in the central carbon metabolism. Additionally, we found that the *sco*AB genes were upregulated, which would suggest that the corresponding enzyme 3-oxoacid CoA-transferase (ScoAB) synthesized acetoacetyl-CoA from succinyl-CoA, which is a part of the succinyl-CoA degradation pathway (not shown). Overall, the differential expression gene profiles suggest a possible NPA degradation pathway, including the subsequent transformation of the majority of the by-products. Following up on this hypothesis, we aimed to confirm the hypothetical degradation pathway using metabolomics and provide more information on PNMR by studying its evolutionary history.

### Pseudomonas sp. Nvir degrades NPA and releases nitrite and nitrate.

**FIG 4:**
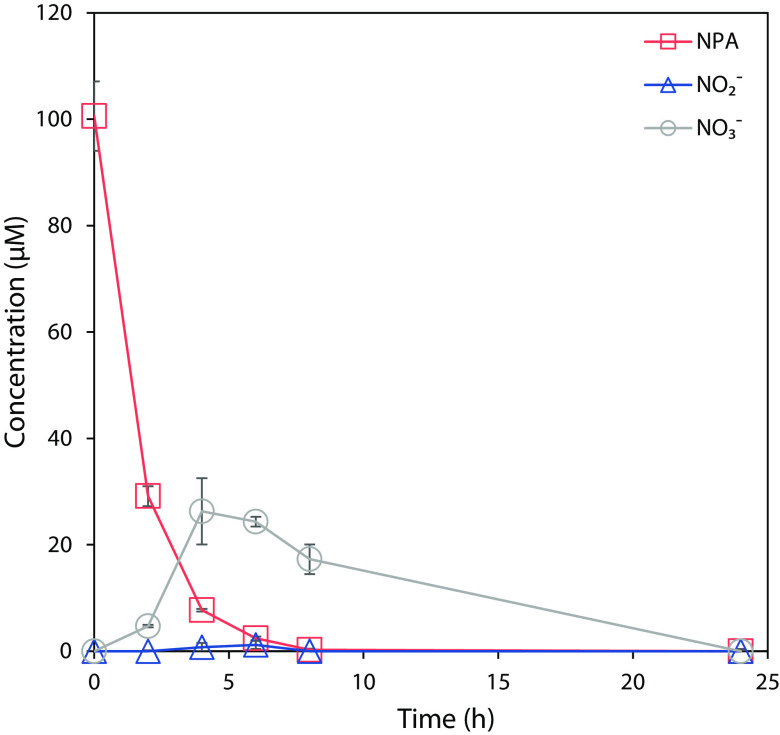
NPA, nitrate, and nitrite profiles in Pseudomonas sp. Nvir cultures. Pseudomonas sp. Nvir precultures were first grown for 24 h in M9 mineral medium with 20 mM glucose and 9.35 mM ammonium chloride as sole carbon and nitrogen source. After the 24-h depletion of the primary substrates, 100 μM NPA was added to the cultures and changes in the concentration of NPA, nitrite, and nitrate were monitored. Data are represented as mean ± standard error (*n* = 3).

We sought to substantiate the proposed NPA degradation pathway by studying the metabolome of Pseudomonas sp. Nvir. Therefore, we aimed to detect metabolic degradation of NPA by the isolate by measuring the concentration of NPA, nitrite, and nitrate. NPA was added to a pregrown culture, and the concentrations of NPA, nitrite, and nitrate were monitored over 24 h (Fig. 4). Within the first 6 h, 100 μM NPA was completely utilized by the culture. As indicated in the transcriptome data, the immediate degradation of NPA was likely caused by the upregulation of already expressed genes when no NPA was present (control samples). Likewise, the constitutive expression of genes involved in NPA degradation with a conceivable low turnover rate of corresponding enzymes would allow rapid utilization of NPA with simultaneous release of nitrite and nitrate.

With this experiment, a steady increase in nitrate and, to a lesser degree, in nitrite concentration was observed soon after NPA supplementation. Likewise, both nitrite and nitrate concentrations gradually decreased after 4 h and were no longer detected at the 24-h time point. As a result, nitrate and nitrite seem to be by-products of the NPA degradation, since in control cultures where no NPA was added, neither nitrate nor nitrite was formed (data not shown). The aforementioned results support the hypothesis that nitrite and nitrate are the products of the proposed NPA breakdown pathway. In addition, the decrease in nitrate and nitrite levels after 4 h suggests that nitrate and nitrite were further transformed and possibly acted as a nitrogen source for Pseudomonas sp. Nvir. This observation is in accordance with the earlier observed growth of Pseudomonas sp. Nvir in the culture supplemented with NPA.

### NPA is used as a nitrogen source.

**FIG 5:**
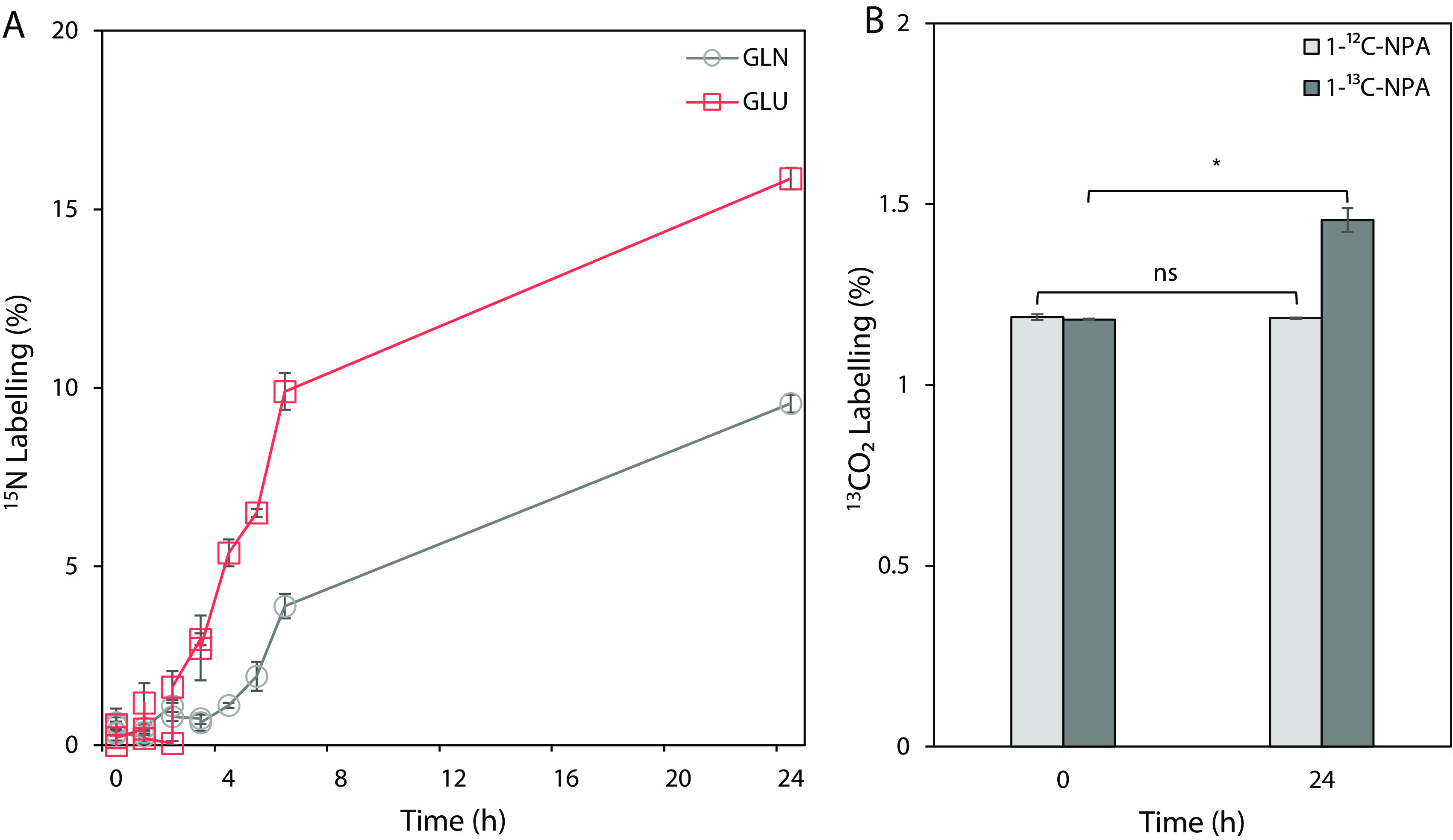
The metabolic fate of ^15^N and ^13^C atoms from the NPA during isotope tracer experiments in Pseudomonas sp. Nvir cultures. (A) ^15^N enrichment of glutamine (GLN) and glutamate (GLU) during [^15^N]NPA tracing experiment. The graph represents the sum of all labeled fractions. (B) ^13^CO_2_ enrichment during the [1-^13^C]NPA tracing experiment. As a control, [1-^12^C]NPA was used. Significance was tested with a Student's *t* test for paired samples and accepted at *P* < 0.05 (*) (*n* = 3); nonsignificant associations are indicated with “ns.” All measured metabolites represent the average of values from three independent biological replicate experiments ± standard error.

To test the hypothesis that nitrate and nitrite present in the medium during NPA degradation were transformed and subsequently used as nitrogen source, an isotope tracing experiment with [^15^N]NPA was performed (Fig. 5A). [^15^N]NPA was synthesized specifically for this study to determine the metabolic fate of the nitrogen atom from NPA during the breakdown reactions. The introduction of the isotopically labeled NPA into the culture revealed the incorporation of ^15^N atoms into glutamine and glutamate. Initially, there was only a small increase in the abundance of [^15^N]glutamine and [^15^N]glutamate, which rapidly increased after 3 h. A simultaneous increase in nitrite and nitrate concentrations reported earlier supports the evidence that inorganic nitrogen by-products were transformed to ammonia and subsequently used in the biosynthesis of amino acids. After 24 h, ~15% of glutamate and 8% of glutamine carried ^15^N atoms. Since glutamine and glutamate are the main nitrogen donating metabolites, these results indicate that NPA acts as a nitrogen source.

### CO_2_ is the by-product of NPA degradation.

To confirm that CO_2_ was generated during the degradation of NPA, we performed a tracing experiment with [1-^13^C]NPA. The results indicated that there was a significant increase in ^13^CO_2_ concentration after a 24-h period (Fig. 5B). No change in the abundance of ^13^CO_2_ was seen in the control. The initial concentration of ^13^CO_2_ in both treated and control samples represents the natural abundance of ^13^CO_2_ in the air. Likewise, the incorporation of ^13^C atoms was not found in amino acids or TCA metabolites, suggesting that CO_2_ is a direct product of NPA degradation. With this experiment, we showed that the carboxyl group was removed from the NPA, which resulted in the release of CO_2_.

### NPA intermediates enter central carbon metabolism.

**FIG 6:**
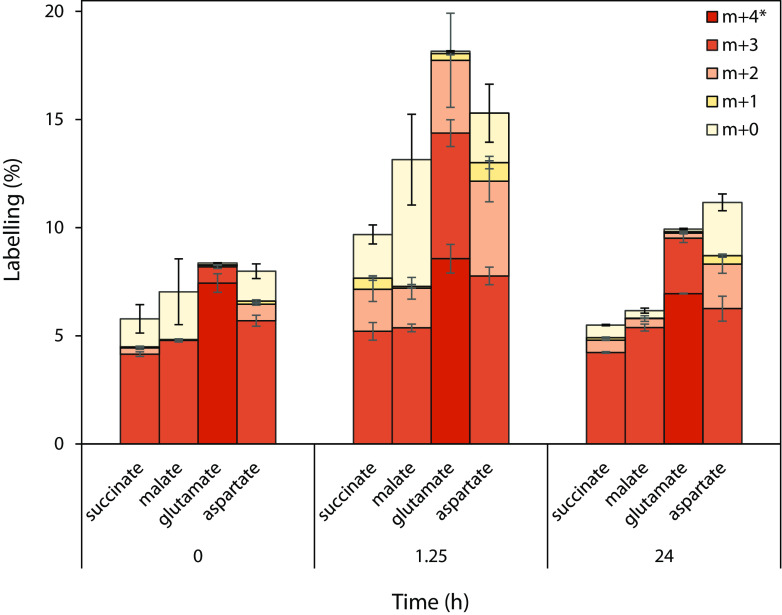
Elucidating the TCA cycle of Pseudomonas sp. Nvir with [U-^13^C]glucose. Time series of the isotope distributions of selected TCA cycle metabolites during isotope tracing experiments. Cells were grown with [U-^13^C]glucose and received a pulse of [U-^12^C]NPA at *t* = 0. The m + 4 fraction is only shown for glutamate. The m + 5 fraction of glutamate and m + 4 fractions of succinate, malate, and aspartate represent the maximum level of labeling, which would obscure the change of the ^13^C label, and are therefore not displayed. The full data set can be found in Fig. S5. All measured metabolites represent the average of three biological replicates ± standard errors.

Ultimately, if our proposed pathway was correct, labeled acetyl-CoA should enter the TCA cycle. To confirm this, we analyzed the labeling profiles of selected TCA cycle metabolites. To do so, we first pregrew Pseudomonas sp. Nvir cultures in [U-^13^C]glucose to uniformly label all metabolites with ^13^C. Next, we fed the biomass with [U-^12^C]NPA and traced the incorporation of ^12^C atoms into succinate, malate, aspartate, and glutamate (Fig. 6). Although aspartate and glutamate are not part of the TCA cycle, they are considered to be in equilibrium with oxaloacetate and α-ketoglutarate, respectively, and therefore mirror their ^12^C labeling. Moreover, we monitored the ^12^C incorporation by comparing the abundance of metabolites in the beginning of the experiment (0 h), after 1.25 h, and after 24 h. The time 1.25 h was selected according to the NPA degradation experiment (Fig. 4) since it was estimated to account for approximately half of NPA being degraded at that point. In this experiment, we looked at the profiles of individual fractions and compared their changes over time.

We hypothesized that two ^12^C atoms from the NPA and correspondingly from the degradation product acetyl-CoA entered the TCA cycle. In accordance with this hypothesis, at 1.25 h an increase in the m + 2 and m + 0 fractions of succinate, malate, and aspartate was observed, with only little change in abundance of the m + 1 fraction. Accordingly, this means that indeed two and four ^12^C carbons, respectively, from acetyl-CoA were incorporated into succinate, malate, and aspartate, generating less-labeled fractions of the aforementioned metabolites. A similar pattern of ^12^C incorporation into glutamate was observed, as illustrated by the increase in the abundance of the m + 3 and m + 2 fractions. Correspondingly, the abundance of all fully labeled fractions of examined metabolites decreased proportionally at 1.25 h.

After 24 h, due to the extensive scrambling of the introduced ^12^C, the abundance of unlabeled fractions of all metabolites decreased and resembled the initial stage. Based on this phenomenon, it seems that ^12^C atoms were first incorporated into the TCA cycle and subsequently, as a result of the metabolite flux, redistributed into various metabolic pathways resulting in an increase in the abundance of fully labeled fractions. These results evidently indicate that the ^12^C from [U-^12^C]NPA was indeed incorporated into the central metabolism and that NPA served as a carbon source.

### *pnm*R is widely distributed among bacteria.

**FIG 7:**
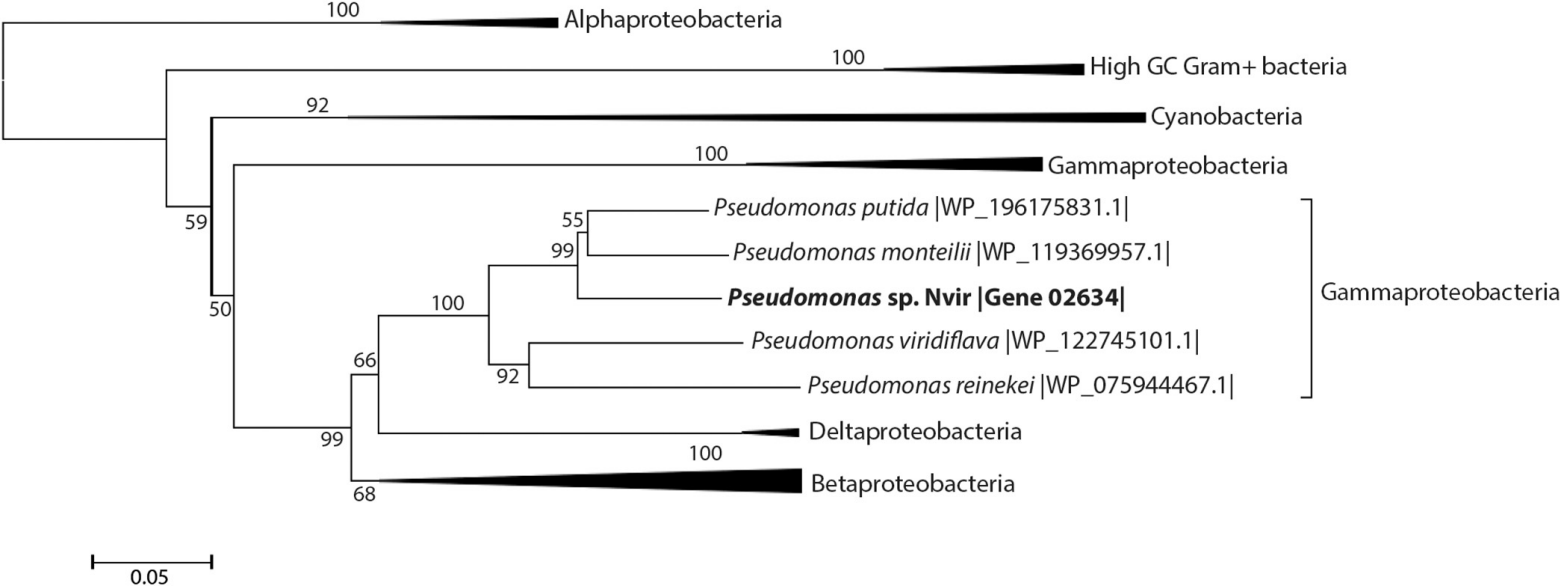
Neighbor-joining tree of *pnm*R homologs found in bacteria. Amino acid sequences were aligned with MUSCLE (49), and phylogenetic trees were calculated with MEGA7 (50) by using 500 bootstrap replicates. The values on the branches are the percentages of replicate trees in which the associated genes clustered together in the bootstrap test for values of ≥50. Branches are drawn to scale, and branch lengths are in the same units as the evolutionary distances used to infer the phylogenetic tree. An unresolved phylogenetic tree is shown in Fig. S6.

We confirmed that Pseudomonas sp. Nvir degrades NPA, and according to the isolate's metabolome, genome, and transcriptome analysis, we established its NPA degradation pathway. To obtain more insight into the distribution of the NPA degradation pathway investigated here, the phylogeny of PNMR was investigated by performing multiple-sequence alignments with proteins clustered within the flavin-dependent monooxygenase family. This protein family is divided into eight groups according to the classification proposed by Huijbers et al. (12). The PNMR amino acid sequence was aligned with representatives of each group, and the similarity was assessed according to the generated alignment. The results showed that PNMR possesses only <63% amino acid similarity to representatives of any of these eight groups (data not shown). Pseudomonas sp. Nvir also contained a classical *pnoA* gene encoding a P3N monooxygenase that clustered with the previously well-characterized Pseudomonas sp. JS189. This gene was not expressed under our experimental conditions and was therefore not responsible for the NPA degradation. Interestingly, PNMR did not cluster with P3N monooxygenase and is therefore evolutionarily distinct, with low sequence similarity (32%). We therefore aimed to assess the distribution of *pnm*R in bacteria by mining the corresponding amino acid sequence in the public database. PNMR as a member of the flavin-dependent oxidoreductase MSMEG 0569 family was found in some *Alphaproteobacteria*, *Betaproteobacteria*, Deltaproteobacteria, *Cyanobacteria*, and bacteria with high G+C content (with minimal amino acid sequence similarity of 40%). The phylogenetic analysis showed that within *Gammaproteobacteria*, PNMR seems to be restricted to the genus Pseudomonas (Fig. 7). Concluding, the analysis demonstrates the low degree of sequence identity of PNMR with other characterized enzymes, in particular P3N monooxygenase, within the flavin-dependent monooxygenase family and establishes its evolutionary distinct position within this family. Furthermore, we show that the *pnm*R gene is widely distributed in many bacteria, suggesting that various bacteria may be capable of NPA degradation.

## DISCUSSION

NPA and its glucose esters are widely distributed naturally occurring nitroaliphatic toxins. They were found to be produced by many leguminous plants, including over 450 different species and varieties of fungi (13, 14). To avert the toxic effect of NPA, nitrotoxin-producing plants and fungi contain enzymes that convert toxic nitroglycosides to nontoxic forms (15). On account of the far-reaching appearance of NPA in nature, many animals, in particular cattle, annelids, and insects, counteract the adverse effects on nitrotoxins by harboring gut-dwelling microorganism degrading NPA. The microbiota of NPA-exposed animals thereby protects their host from intoxication. So far, the ability to biodegrade nitrotoxins had been shown to be carried out via different enzymatic routes.

In the rumen of cattle, NPA glucose conjugates are rapidly hydrolyzed, liberating free NPA, which subsequently is either further metabolized by the ruminal microbiota to β-alanine and aminopropanol or absorbed into the circulatory system (16). Along these lines, the bacterial isolate from the gut of the earthworm Eisenia fetida (Opisthopora: Lumbricidae) was found to grow exclusively on NPA as the sole carbon, nitrogen, and energy source (17). Contrarily, yet only hypothesized, a partial microbial role in NPA degradation was suggested in grasshoppers (Melanoplus bivittatus and *M. sanguinipes*; Orthoptera: Acrididae), where NPA detoxification occurs by the conjugation of the NPA with amino acids and subsequent excretion of NPA amides by the insect (18). Nonetheless, bacteria isolated from other ecological niches were able to degrade NPA too. Similar to the earthworm isolate, bacteria obtained from soil and water samples grew aerobically on NPA as the sole carbon, nitrogen, and energy source. As a result of NPA degradation, 3OP, nitrite, nitrate, and hydrogen peroxide were formed (8).

In this study, we investigated the biodegradation pathway of NPA of a Pseudomonas sp. Nvir isolated from the gut of the pest insect *N. viridula*. The results indicate that both nitrate and nitrite were intermediary products that were transformed into ammonium and subsequently used as building blocks for the biosynthesis of amino acids. The isotopologue tracer experiment with [^15^N]NPA showed the enrichment of [^15^N]glutamine and [^15^N]glutamate over time, which indicates that NPA serves as a nitrogen source. Furthermore, CO_2_ was released from the carboxylic acid group of NPA, yielding a two-carbon by-product, which was further utilized in the central carbon metabolism of Pseudomonas sp. Nvir through the TCA cycle. In addition, the transcriptome analysis suggested that one of the transformation products of 3OP was β-alanine, indicating that the NPA metabolism by Pseudomonas sp. Nvir was somewhat similar to that of rumen bacteria, where accumulation of β-alanine was observed during NPA degradation (1).

The biochemical fate of NPA in eukaryotes and bacteria has been extensively studied, and the substantial differences within the NPA degradation pathways are now apparent. Yet, there still seems to be knowledge gaps in the characterization of the genes and enzymes involved in the degradation pathway. Currently, many identified enzymes involved in the NPA degradation are orphan enzymes (19). The first crucial step in the degradation, however, seems to rely on flavoproteins which have oxidoreductive activity. Nitrotoxin-synthesizing plant and fungi produce various enzymes, such as NPA oxidase (NPAO), P3NO, NMO, and nitroalkane oxidase (NAO), which transform NPA and its conjugate base to numerous intermediates (7–9, 15, 20–22). Likewise, an NPA-degrading Pradoshia eiseniae gen. nov., sp. nov. strain isolated from the earthworm’s gut encoded two homologous NMOs in the genome. Interestingly, Pseudomonas sp. JS189 harbored P3N monooxygenase encoded by *pnoA*, whereas in P. aeruginosa PAO1 NPA degradation was catalyzed by NMO encoded by *nmoA*. This demonstrates that even bacteria within the same genus may produce different NPA-degrading enzymes. In this study, we observed that the previously unidentified gene *pnm*R was upregulated in Pseudomonas sp. Nvir under conditions in which NPA was plentiful. Based on its phylogeny and metabolomic analysis, we hypothesized that the corresponding PNMR of Pseudomonas sp. Nvir hence harbors a similar catalytic activity to the only distantly related bacterial P3N monooxygenase and is a key enzyme in NPA degradation. It is conceivable that the enzymes similarly act upon NPA degradation as they both release nitrate and nitrite as the intermediate degradation products. Interestingly, NMO and NAO release no nitrate while degrading NPA (8). Ultimately, to reveal whether PNMR is indeed a nitronate monooxygenase, further isolation of the enzyme and its biochemical characterization will be required.

The functional characterization of the enzymes involved in the NPA degradation may provide insights into the bioconversion of NPA-contaminated products and could give rise to multiple applications in industry. As reported by Torres-Guzman et al., nitrotoxin-degrading enzymes could be applied in biotechnology (10). Nitronate monooxygenases are broadly distributed enzymes that have diverse catalytic efficiencies and preferred substrates, which allows them to be used as pharmaceutical agents, biocatalysts, and in bioremediation. Therefore, the characterization of PNMR found in Pseudomonas sp. Nvir might be a new means of reducing the negative impact of NPA in many important industrial sectors.

To conclude, the results of this study revealed that the *pnm*R gene could encode an enzyme for NPA degradation. By using an array of complementary techniques, we shed light on the metabolic potential of Pseudomonas sp. Nvir and established its degradation pathway. With this study, we therefore provide insights into the microbial degradation and tolerance mechanisms toward toxic nitroglycosides, which suggests possible applications of bacteria from understudied ecosystems in the industry.

So far, degrading bacteria have been found to be acquired by insects to perform symbiont-mediated detoxification, through which insects gain protection against toxic plant metabolites and insecticides (23). Currently, the role of the Pseudomonas sp. Nvir in the Southern green shield bug remains unclear, and from our research project, it is not possible to deduce whether the isolate is a mutualistic symbiont in the insect’s gut microbiome or whether it is a transient environmental bacterium. It is, however, tempting to speculate that like other gut-dwelling microorganism, the NPA-degrading Pseudomonas sp. strain Nvir might serve its host by protecting it against the toxic influence of nitroglycosides.

## MATERIALS AND METHODS

### Insect rearing.

Nezara viridula (42 insects, 2nd through 4th instar nymphs) were collected in the field from creeping thistle (Cirsium arvense) in The Netherlands (51.348028, 6.128802) on 5 July 2019. The insects were transferred to the greenhouse and placed within the rearing cage to establish a colony. *N. viridula* individuals were reared in a greenhouse facility with normal daylights and additional light to obtain a photoperiod of 16 h/8 h (light/dark) year-round. Insects were provided with sunflower (Helianthus annuus), soybean (Glycine max), and brown mustard (Brassica juncea) seeds and native plants of crown vetch (Securigera varia), black mustard (Brassica nigra), and black nightshade (Solanum nigrum).

### Bacterial strain isolation.

Ten complete gut systems (V1 to V4 sections) of adult *N. viridula* insects were dissected, pooled, and mixed in a tube. Gut tissue was disrupted by adding beating beats to a tube and 200 μL of sterile phosphate-buffered solution (137 mM NaCl, 2.7 mM KCl, 10 mM Na_2_HPO_4_, 1.8 mM KH_2_PO_4_) and brief vortexing. One hundred microliters of gut suspension was added to 10 mL of M9 mineral salt medium (33.7 mM Na_2_HPO_4_, 22 mM KH_2_PO_4_, 8.55 mM NaCl, 9.35 mM NH_4_Cl, 1 mM MgSO_4_, 0.3 mM CaCl_2_, Thauer vitamin mixture [24], trace elements [25], pH 7.2) supplemented with 100 μM NPA (Merck, Germany) as a carbon source. Cultures were grown aerobically in Erlenmeyer flasks (500 mL) at 30°C and 200 rpm. To obtain pure cultures of NPA-degrading bacteria, pregrown cultures were streaked onto an M9 mineral salt agar plate supplemented with 100 μM NPA. Several colonies were picked and restreaked three times onto fresh M9 agar plates. Isolated pure cultures were incubated in M9 mineral medium deprived of ammonium chloride, with 100 μM NPA as the sole nitrogen and carbon source, in a manner similar to that described above. To define the purity of the cultures which had grown on NPA as a sole nitrogen and carbon source, DNA was extracted and the genomes were sequenced with Mi-Seq Illumina sequencing (Illumina, San Diego, CA, USA) as described below.

### Genome sequencing and data analysis.

DNA was extracted from NPA-degrading pure cultures using the DNeasy PowerSoil kit (Qiagen, Germany) according to the manufacturer’s protocol, using 1 mL pelleted cell culture lysed with the TissueLyser LT (Qiagen) for 2 min at 50 Hz. For the genome of the bacterial isolates, the DNA libraries were prepared with the Nextera XT library preparation kit (Illumina, San Diego, CA, USA) according to the manufacturer's instructions. The libraries were checked for quality and size distribution using the Agilent 2100 bioanalyzer and a high-sensitivity DNA kit (Agilent Technologies, Santa Clara, CA, USA). Quantitation of the libraries was performed by the Qubit double-stranded DNA (dsDNA) HS assay kit (Thermo Fisher Scientific, Inc., Waltham, MA, USA). Paired-end sequencing (2× 300 bp) was performed using the Illumina MiSeq sequencer (Illumina, San Diego, CA, USA) and the MiSeq reagent kit v3 (Illumina) according to the manufacturer's protocol.

The quality of Illumina paired-end genomic sequencing data was assessed using FASTQC 0.11.8 (26) before and after quality processing. Quality trimming, adapter removal, and contaminant filtering of reads were performed using BBDuk (BBTools 38.75) (26). Trimmed reads were coassembled *de novo* using MEGAHIT 1.2.9 (27). MEGAHIT assembled the genome using k-mer sizes of 21, 29, 39, 59, 79, 99, 119, and 141. Reads were mapped back to the genomes using separately BBMAP 38.75 (default settings), Bowtie 2 2.3.5, or Burrows-Wheeler Aligner (BWA MEM) 0.1.17 (26, 28). The sequencing mapping files were handled and converted using SAMtools 1.10 (29). Genome binning was performed for contigs greater than 1,500 bp using the four binning algorithms BinSanity 0.3.1 (30), CONCOCT 1.1.0 (31), MaxBin2 2.2.7 (32), and MetaBAT 2 2.15 (33) with default settings. The bin sets were supplied to DAS Tool 1.1.2 (34) for consensus binning to obtain the final optimized bins. Out of all sequenced reads, <100 reads were not mapped to the genome bins. The quality of the generated bins was assessed through a single-copy marker gene analysis with CheckM 1.1.2 (11). Taxonomic assignment for the trimmed sequencing reads and assembled genomes was performed with Kaiju (35). The genomes were automatically annotated with Prokka 1.13.4 (36). Genome annotations were examined using Artemis 16.0.0 (37). Additional genome information is found in Table S1 in the supplemental material and represents only features of the Pseudomonas sp. Nvir bin.

The analysis of average amino acid identity (AAI) and average nucleotide identity (ANI) of Pseudomonas sp. Nvir was executed with tools from http://enve-omics.ce.gatech.edu/ in 2021 as described previously (38, 39). AAI and ANI matrices were calculated based on best hits (one-way AAI and ANI) and reciprocal best hits (two-way AAI and ANI) between genomic data sets.

### Transcriptome sequencing and data analysis.

Prior to RNA extraction, the Pseudomonas sp. Nvir culture grew in an M9 mineral salt medium supplemented with glucose and ammonium as described earlier. Upon C/N depletion, NPA was added to treated cultures. Biomass samples were collected at 1.25 h after the supplementation of NPA. The time 1.25 h was selected according to the NPA degradation experiment (Fig. 4) since it was estimated to account for approximately half of the NPA being degraded at that point. Control biomass samples, where no NPA was added, were collected at the same time point. Two-milliliter samples were centrifuged at 20,000 × *g* for 3 min at 4°C, and RNA was extracted with the RiboPure-Bacteria kit (Thermo Fisher Scientific) according to the manufacturer's instructions. Prior to sequencing, bacterial mRNA was purified with the MICROBExpress bacterial mRNA enrichment kit (Thermo Fisher Scientific) according to the manufacturer's instructions. Transcriptome libraries were constructed using the TruSeq stranded mRNA library prep protocol (Illumina) according to the manufacturer’s instructions. Obtained libraries were checked with Qubit as described before. Equimolar pooled libraries were sequenced using the Illumina MiSeq sequencer (Illumina). For sequencing, the 150-bp sequence chemistry was performed using the MiSeq reagent kit v.3 (Illumina) according to the manufacturer's protocol in one direction. For both control and treated cultures, RNA was extracted from three independent biological replicates.

The quality of Illumina single-end transcriptome raw reads was assessed with FASTQC 0.11.8 (26) prior to read mapping. Single-end reads were mapped against the annotated genome (Prokka 1.13.4) using Kallisto 0.46.2 (40), and outputs were summarized with MultiQC 1.11 (41). Read counts were transformed in Tximport (42) for differential gene expression analysis using BioConductor packages in the R environment (43). To determine differentially expressed genes, a log_2_ fold change cutoff of 1 was set. In addition, to address the issue of multiple testing, a false-discovery rate (FDR) correction was performed with a *P* value of *<*0.01 using the Benjamini-Hochberg method (44). The differential gene expression was compared between the NPA-supplemented cultures and control cultures, where no NPA was added. The heat maps illustrating differentially expressed gene profiles were constructed in the R environment using the heatmap.2 package and displayed as the average of three independent biological replicate experiments.

### Cultivation of Pseudomonas sp. Nvir and sample collection.

The axenic culture of Pseudomonas sp. Nvir was pregrown in batch cultivation (30°C, 200 rpm) with M9 medium containing glucose (20 mM) and ammonium (9.35 mM). After nitrogen and carbon source depletion, 100 μM NPA was supplemented. Afterward, 2× 1 mL of sample (supernatant and cell pellet, obtained by centrifugation at 20,000 × *g*, 3 min) were collected for the determination of NPA degradation, glucose, ammonium, nitrite, and nitrate concentrations (supernatant), and culture growth (cell pellet). Supernatant and cell pellet samples were stored at −20°C until analysis. Samples were collected at 0, 2, 4, 6, 8, and 24 h after NPA addition.

### Growth of Pseudomonas sp. Nvir.

Growth of Pseudomonas sp. Nvir on NPA as a carbon, nitrogen, and energy source was followed by measuring the optical density at 600 nm (OD_600_) with a Cary 60 UV-visible (UV-Vis) spectrophotometer (Agilent Technologies, USA) for 196 h. The experiment was carried out with three independent biological replicates.

### Chemical and protein quantification. (i) Nitropropionic acid.

High-performance liquid chromatography (HPLC) was performed on an Agilent 1100 system equipped with a diode array detector and a Merck C_18_ column (LiChrospher 100 RP-18 end-capped [5-μm] column, 250 mm by 4.6 mm). Isocratic analysis was performed with 100% 0.1% *ortho*-phosphoric acid in water with a flow rate of 1.2 mL min^−1^. Prior to the analysis, 200 μL of supernatant was acidified with 25 μL 1 M sulfuric acid and 100 μL of the sample was injected. NPA was measured at 210 nm with a retention time of 5.2 min.

### (ii) Glucose.

The glucose concentration in the cultures’ supernatant was measured with a glucose colorimetric/fluorometric assay kit (Merck) according to the manufacturer's protocol.

### (iii) Ammonium.

Ammonium concentrations were determined with the *ortho*-phthaldialdehyde (OPA) method as described previously (45).

### (iv) Nitrite and nitrate.

To determine the NO_2_^−^ and NO_3_^−^ concentrations, the Griess reagent assay was used. One hundred microliters of supernatant was transferred to a 96-well plate containing 100 μL of Griess reagent, consisting of 50 μL of reagent A (1% [wt/vol] sulfanilic acid in 1 M HCl) and 50 μL of reagent B (0.1% [wt/vol] naphthylethylene diamine dihydrochloride in water). The plate was incubated for 10 min at room temperature, and absorbance was measured at 540 nm with a microplate reader (SpectraMax 190; Molecular Devices, San Jose, CA, USA). Next, 27 μL of VCl_3_ (10 mg mL^−1^ in 1 M HCl) was added to the sample to reduce nitrate to nitrite. Subsequently, the 96-well plate was then incubated in the dark at 60°C for 30 min, and afterward, absorbance was measured at 540 nm. NaNO_3_ and NaNO_2_ were used to prepare standard curves.

### (v) Protein quantification.

Cell growth was determined by measuring the protein concentration of a 200-μL cell pellet with a Pierce bicinchoninic acid (BCA) protein assay kit (Merck).

### Synthesis of labeled [^15^N]- and [1-^13^C]nitropropionic acid.

Stable isotope-labeled [^15^N]- and [1-^13^C]NPA were synthesized according to reference 5 with modifications. In summary, for [^15^N]NPA the starting material 3-bromopropionic acid was added to a stirred suspension of sodium [^15^N]nitrite in dry dimethylformamide (DMF) and the solution was stirred at room temperature for 40 h. The reaction mixture was diluted with water, adjusted to pH 1 with a 1 M aqueous HCl solution and extracted with diethyl ether.

For [1-^13^C]NPA, the starting material 3-bromopropanoic-1-^13^C acid was added to a stirred suspension of sodium nitrite in dry DMF, and the solution was stirred at room temperature for 28 h. The reaction mixture was diluted with water adjusted to pH 1 with a 1 M aqueous HCl solution and extracted with ethyl acetate.

Organic layers of both labeled NPA were washed with brine (saturated NaCl aqueous solution), dried over MgSO_4_, and evaporated *in vacuo*. The crude product was purified using column chromatography (ethyl acetate-heptane, 0% to 100%). The ^1^H NMR and ^13^C NMR spectra were recorded at 298 K on a Bruker Avance III 400 (400 MHz) or 500 (500 MHz) spectrometer. The detailed synthesis protocol and NMR spectra are included in the supplemental material.

### Isotope tracing experiments.

Isotope tracing experiments with [^15^N]- and [1-^13^C]NPA were performed in batches with 30 mL M9 medium in 120-mL culture bottles, which were sealed with crimp-capped butyl rubber stoppers. In all isotope tracer experiments, 100 μM NPA was supplied to the cultures. After [^15^N]NPA supplementation, 500-μL samples were rapidly withdrawn at time points 0, 0.25, 0.5, 0.75, 1, 1.25, 1.5, 1.75, 2, 2.5, 3, 3.5, 4, 5, 6, and 24 h. In the [1-^13^C]NPA experiment, samples were only collected at the 0- and 24-h time points to minimize the potential loss of ^13^CO_2_ caused by frequent sampling. For the reverse isotope tracing experiment with U-^13^C isotopically labeled glucose and [U-^12^C]NPA, a Pseudomonas sp. Nvir culture was pregrown for 24 h on [U-^13^C]glucose (Cambridge Isotope Laboratories, Tewksbury, MA, USA) and passaged three times to cultivate highly ^13^C-enriched biomass. Next, the culture was exposed to [U-^12^C]NPA, and samples were collected at time points 0, 1.25, and 24 h. All experiments were carried out in three biological replicates, and pelleted cell cultures obtained by centrifugation (20,000 × *g*, 3 min) were stored at −70°C until further analysis.

Prior to isotope tracing experiment analysis, the extraction of biomass was performed with a mixture of acetonitrile, methanol, and H_2_O (40:40:20 [vol/vol/vol]). In short, 200 μL of the 40:40:20 mixture was added to the pelleted cells. Samples were briefly vortexed and incubated for 5 min on ice. Next, samples were centrifuged (20,000 × *g*, 5 min, 4°C) and the supernatant was transferred into a new tube and stored at −70°C until metabolite analysis. Samples were analyzed with a 1290 Infinity II liquid chromatography (LC) system coupled to a 6546 quadrupole time of flight mass spectrometer (Agilent Technologies) (LC/Q-TOF MS) as described previously (46). In short, 2 μL sample was injected onto a Diamond hydride type C column (Cogent) and separated using a 0.4-mL min^−1^ gradient of water with 0.2% formic acid (A) and acetonitrile with 0.2% formic acid (B). The gradient was as follows: 0 to 2 min of 85% B, 3 to 5 min of 80% B, 6 to 7 min of 75% B, 8 to 9 min of 70% B, 10 to 11 min of 50% B, 11 to 14 min of 20% B, and 14 to 24 min of 5% B, followed by 10 min of reequilibration at 85% B. Detection of compounds was performed in the positive- and negative-ionization modes.

A custom library composed of accurate masses and LC retention times of central carbon and nitrogen metabolites were used to explore the core metabolome of Pseudomonas sp. Nvir. The relative abundance of metabolites was determined using Agilent Qualitative Analysis 10.0 and Profinder 10.0 tools. Isotopologue distribution analysis of selected metabolites during isotope tracing experiments was conducted based on the relative isotope intensities. [^15^N]- and [1-^13^C]NPA samples were corrected for the natural ^13^C and ^15^N abundance by Profinder 10.0. This correction was not applicable for the reverse labeling experiment, for which we used uncorrected peak areas.

### Gas chromatography-mass spectrometry analysis of dissolved inorganic carbon.

Isotopic fractions of dissolved inorganic carbon (DIC) in the liquid medium were measured based on a modified headspace method (47). One milliliter of liquid culture was collected from the batch incubations with a syringe, directly filtered through a sterile 0.45-μm-pore filter (Whatman; cellulose acetate) and a 26G needle into a vial (12 mL) (Exetainer; Labco, Ltd., United Kingdom) containing 340 μL 6 M HCl, and crimp sealed with a rubber stopper. Prior to addition of the liquid sample, vials with HCl were flushed with 100% N_2_ gas to void the headspace of background CO_2_. Samples were equilibrated with the acid in the bottles for at least 1 h at room temperature to drive all DIC into the gas phase. Fifty microliters of the bottles’ headspace was injected into a gas chromatograph (Agilent 6890 equipped with a 6-ft Porapak Q column) coupled to a mass spectrometer (Agilent 5975C MSD; Agilent, Santa Clara, CA) with a gas-tight syringe (Hamilton, Reno, NV, USA). The gas chromatograph was set at 80°C with helium as a carrier gas at a flow rate of 24 mL min^−1^ to determine the isotopic fraction of ^13^CO_2_.

### Phylogeny analysis.

To determine the distribution of the *pnm*R gene product, the PNMR amino acid sequence was used as a query in a BLASTp search at NCBI (48) that targeted only bacteria (taxid 2). The hits with a percentage of identity of >40% and E values of <1.0 × e−20 were considered in the further analysis. Likewise, the phylogeny of the *pnm*R was assessed within the flavin-dependent monooxygenase family, based on the classification (12). The amino acid sequences for representative proteins within every class were obtained from UniProt (UniProt, 2021). The amino acid sequences were extracted and compared by multiple sequence alignment with MUSCLE v.3 8.31 (49), and phylogenetic trees were calculated with MEGA7 (50). The evolutionary history was inferred by the neighbor-joining method with 500 bootstraps.

### Data availability.

The transcriptome and genome sequencing data have been deposited in the European Nucleotide Archive under accession no. PRJEB49126.
