# Supplementary material for: Insect Gut Isolate Pseudomonas sp. Strain Nvir Degrades the Toxic Plant Metabolite Nitropropionic Acid
Source: Appl Environ Microbiol. 2022 Sep 26;88(19):e00719-22. doi: 10.1128/aem.00719-22 (PMC9552603; doi:10.1128/aem.00719-22)
Supplement: Supplemental file 1 — Supplemental material. Download aem.00719-22-s0001.pdf, PDF file, 1.4 MB [file aem.00719-22-s0001.pdf]

Supplementary material

Supplementary Table 1. Summary of the *Pseudomonas* sp. Nvir genome features. Genome of *Pseudomonas* sp. Nvir was sequenced with Illumina MiSeq Sequencer. Genome binning was performed for contigs greater than 1500 bp. The genomes were automatically annotated with Prokka 1.13.4.

**Genome size (bp)**	5716865
**Completeness**	99.34%
**Contamination**	1.85%
**Strain heterogeneity**	0
**Contigs**	210
**Plasmids**	Unknown
**Total number of genes**	5464
**CDS**	5320
**rRNAs**	4
**tRNAs**	70
**tmRNAs**	1
**G+C content**	62.3%

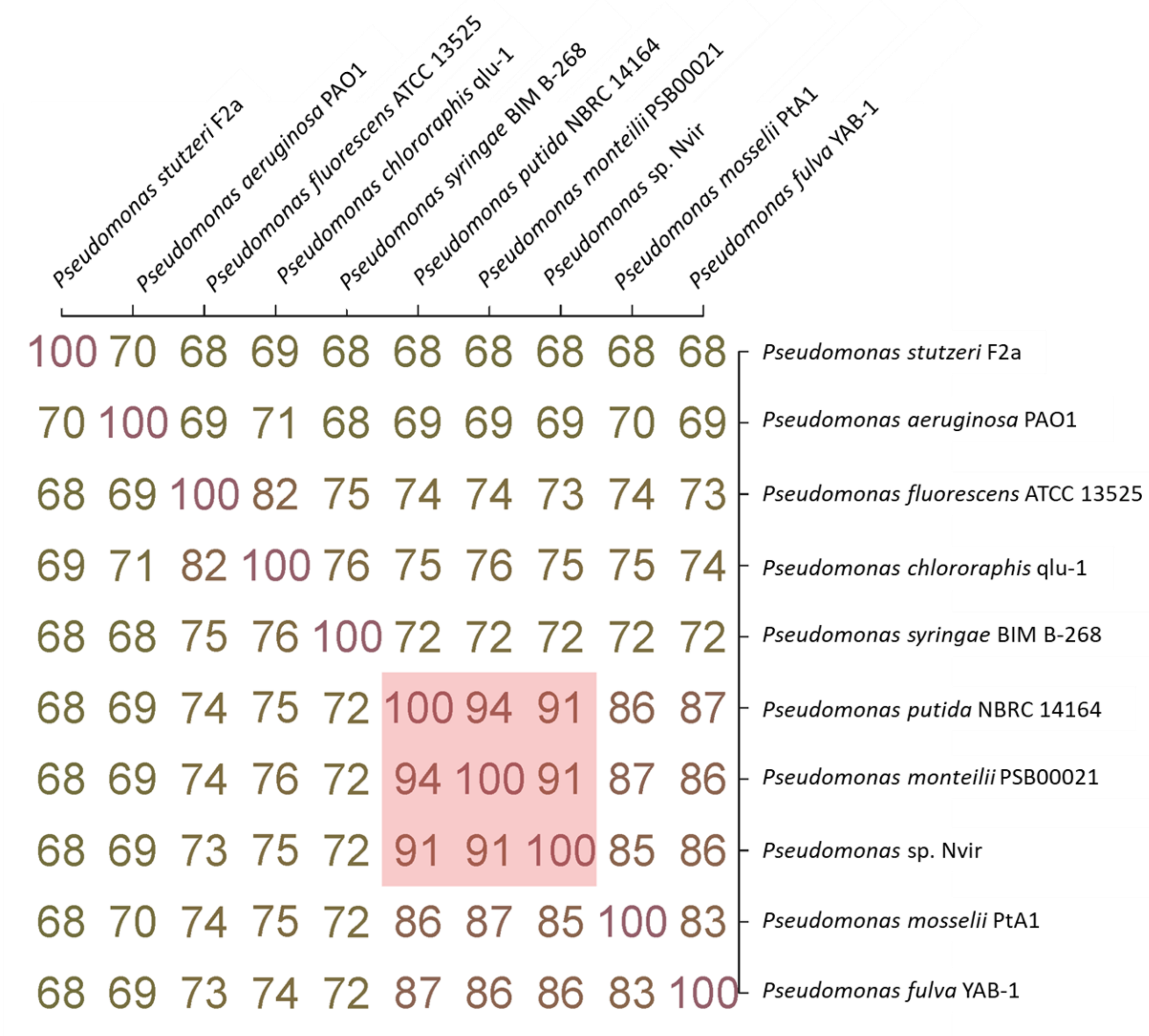

Supplementary Figure 1. The analysis of Average Amino acid Identity (AAI) of *Pseudomonas* sp. Nvir. AAI analysis was executed with online tool (http://enve-omics.ce.gatech.edu/, 2021) as described (Goris, Konstantinidis et al. 2007, Rodriguez-R and Konstantinidis 2016). Briefly, ten genomes of *Pseudomonas* sp. genuses were obtained from NCBI database and aligned together with *Pseudomonas* sp. Nvir of this study. AAI matrix was calculated based on best hits (one-way AAI) and reciprocal best hits (two-way AAI) between genomic datasets.

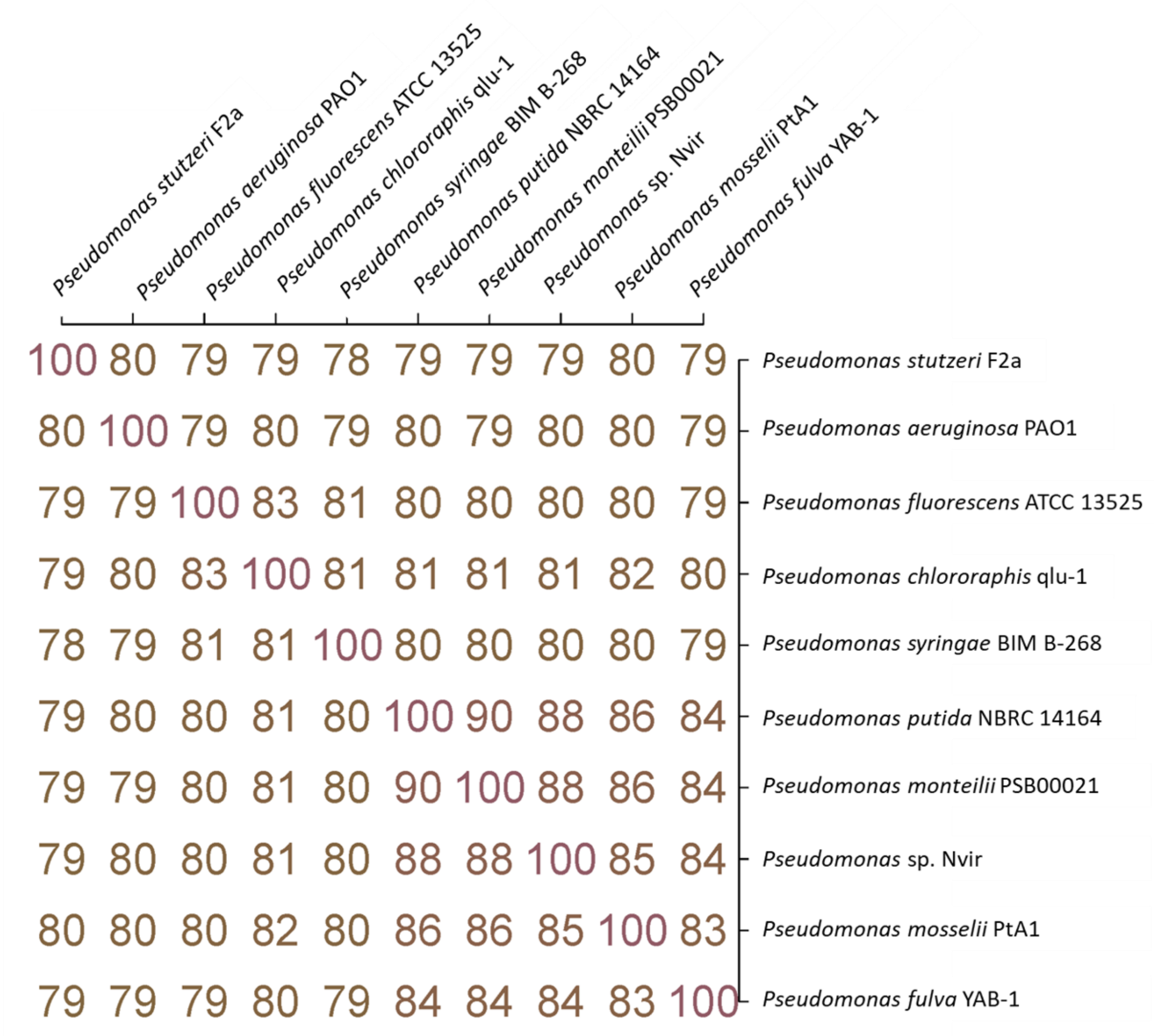

Supplementary Figure 2. The analysis Average Nucleotide Identity (ANI) of *Pseudomonas* sp. Nvir. ANI analysis was executed with online tool (http://enve-omics.ce.gatech.edu/, 2021) as described (Goris, Konstantinidis et al. 2007, Rodriguez-R and Konstantinidis 2016). Briefly, ten genomes of *Pseudomonas* sp. genuses were obtained from NCBI database and aligned together with *Pseudomonas* sp. Nvir of this study. ANI matrix was calculated based on best hits (one-way ANI) and reciprocal best hits (two-way ANI) between genomic datasets.

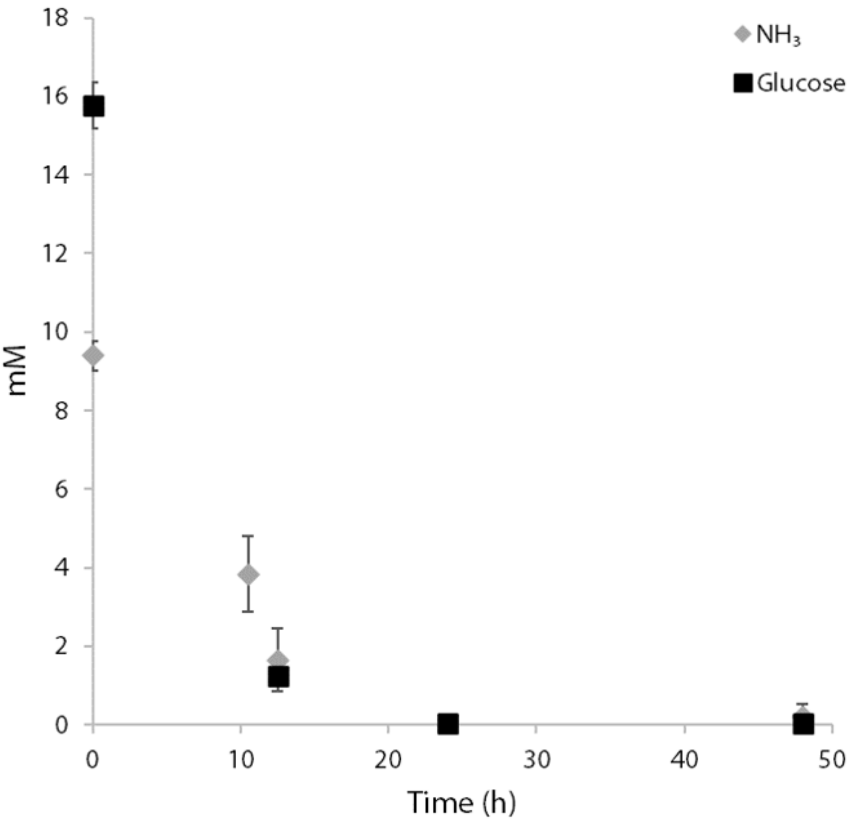

Supplementary Figure 3. Glucose and ammonium measurements over 48 hours in *Pseudomonas* sp. Nvir cultures. The medium was inoculated with approximately 20 µg protein/mL biomass and pre-grown in an M9 mineral medium containing glucose and ammonium. Subsequently, *Pseudomonas* sp. Nvir cultures were grown for 24 hours upon glucose and ammonia were depleted from the medium. Glucose and ammonium concentrations were also monitored at 48h. Data are represented as mean +/- standard error (n=3).

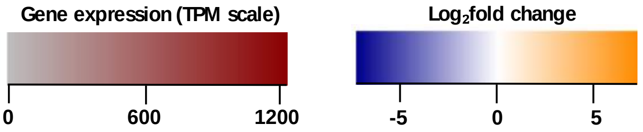

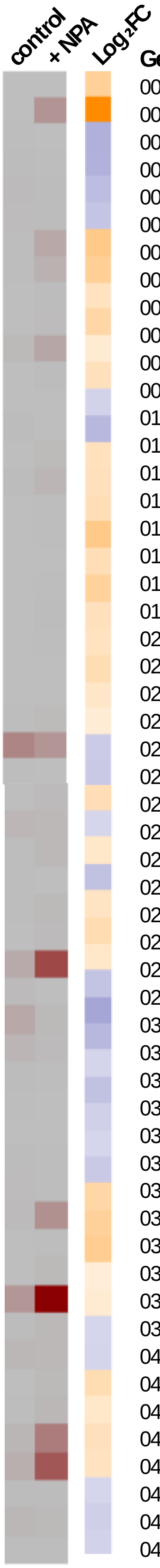

Gene nr	Gene	Predicted product
00035	*nas* D	Nitrite reductase [NAD(P)H]
00108	*hmp*	Flavohemoprotein
00145	*mdl* C	Benzoylformate decarboxylase
00146	*pca* K1	4-Hydroxybenzoate transporter PcaK
00147	*pch* A	4-Hydroxybenzaldehyde dehydrogenase (NADP(+))
00152	*pan* E1	2-Dehydropantoate 2-reductase
00437	*sco* A	Putative succinyl-CoA:3-ketoacid conenzyme A transferase subunit A
00438	*sco* B	Putative succinyl-CoA:3-ketoacid conenzyme A transferase subunit B
00690	*rcs* C2	Sensor histidine kinase RcsS
00787	*dad* A1	D-amino acid dehydrogenase
00850	-	Hypothetical protein
00999	*yci* O	Putative protein YciO
01146	*pil* Q	Type IV pilus biogenesis and competence protein PilQ
01252	-	Omega-amino acid-pyruvate aminotransferase
01253	*bau* C1	Putative 3-oxopropanoate dehydrogenase
01329	*dus* B	tRNA-dihydrouridine synthase B
01485	*qed* A1	Quinoprotein alcohol dehydrogenase (cytrochrom C)
01487	-	Hypothetical protein
01842	*pha* A	Acetyl-CoA acetyltransferase
01971	-	Putative HTH-type transcriptional regulator
02096	*nan* T	Sialic acid transporter NanT
02128	*fcu* A1	Ferrichrome receptor FcuA
02232	-	Hypothetical protein
02281	*acs* A2	Acetyl-CoA synthetase
02331	*inf* C	Translation initiation factor IF-3
02385	-	Hypothetical protein
02492	*mmsA*/*iol* A	Methylmalonate-semialdehyde dehydrogenase (acelating)
02634	*pnm* R	Putative nitronate monooxygenase [reductase]
02518	*all* A	Ureidoglycolate lyase
02641	*nor* R2	Anaerobic nitric oxide reductase transcription regulator NorR
02667	*int* S	Prophage integrase IntS
02739	*rpp* H	RNA pyrophosphohydrolase
02784	-	Hypothetical protein
02996	*gro* S	10 kDa chaperonin
03052	-	Hypothetical protein
03056	*hag* 055	Flagellin
03058	-	Ubiquinone biosynthesis O-methyltransferase
03060	-	Hypothetical protein
03338	-	Hypothetical protein
03344	-	Hypothetical protein
03566	-	Hypothetical protein
03707	-	Hypothetical protein
03779	*rfn* T	Riboflavin transporter RfnT
03780	-	Hypothetical protein
03826	*yoh* C	Inner membrane protein YohC
03964	*fdo* H	Formate dehydrogenase-O iron-sulfur subunit
04103	*pqiA* 2	Intermembrane transport protein PqiA
04584	-	Hypothetical protein
04732	*cta* G	Cytochrome C oxidase assembly protein CtaG
04762	-	Hypothetical protein
04764	*ivy*	Inhibitor of vertebrate lysozyme
04987	*pot* A5	Spermidine/putrescine import ATP-binding protein PotA
04988	*pot* D	Spermidine/putrescine-binding periplasmic protein
04989	-	Hypothetical protein

Supplementary Figure 4. *Pseudomonas* sp. Nvir differential gene expression profile under NPA conditions. *Pseudomonas* sp. Nvir cultures grew in an M9 mineral medium supplemented with glucose and ammonium. After 24h C/N depletion phase, 100 µM of NPA was added to the culture. Samples for the RNA extraction were collected at 1.25h after NPA supplementation. Likewise, control samples where no NPA was added, were collected at the same time point. A heatmap shows transcript expression level (TPM) under presence (+ NPA) or absence of NPA (control) and Log_2fold changes in the gene expression in the ten differentially expressed genes in *Pseudomonas* sp. Nvir.

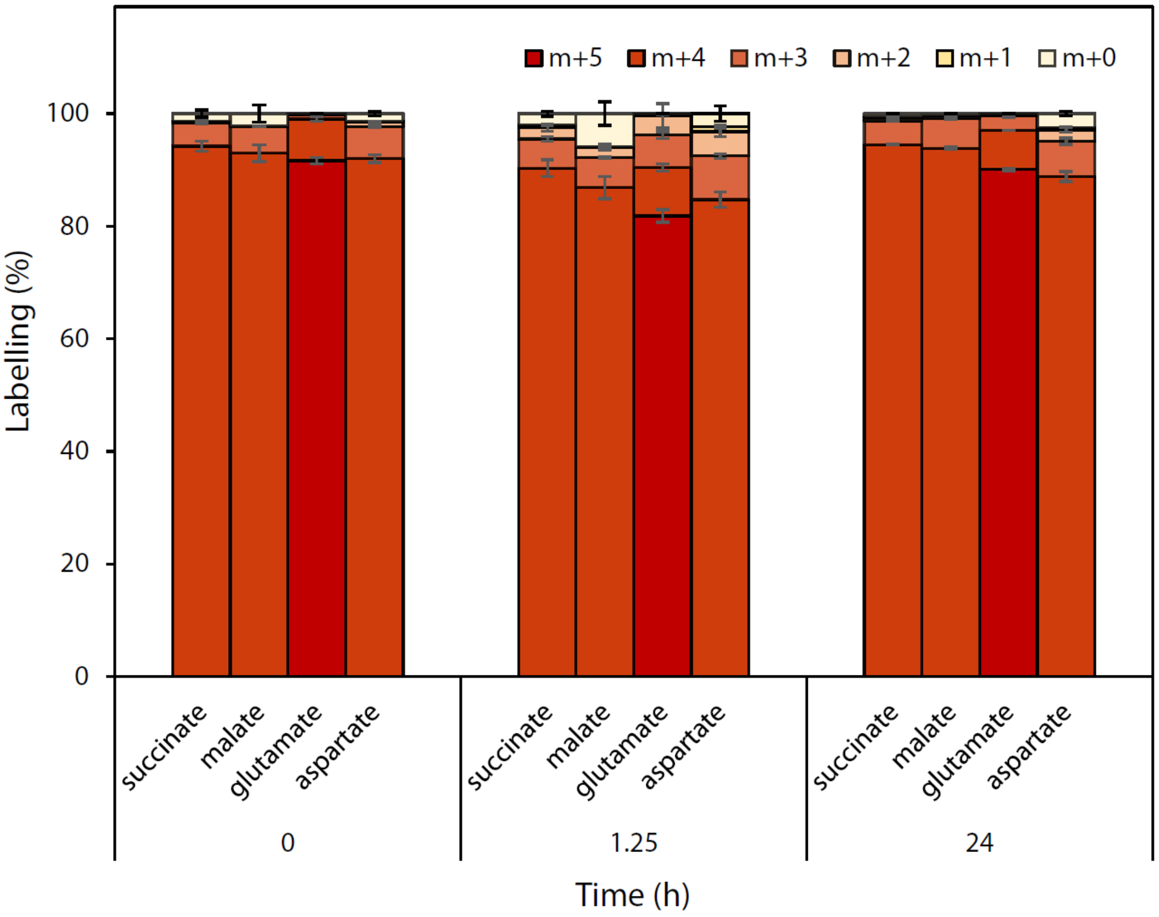

Supplementary Figure 5. Elucidating the TCA cycle of *Pseudomonas* sp. Nvir with U-^{13}C-glucose. Time-series of the mass isotopologue distributions of selected TCA cycle metabolites during isotope tracer experiments. Cells were grown with U-^{13}C-glucose and received a pulse of U-^{12}C-NPA at t=0. The m+5 fraction of glutamate and m+4 fractions of

succinate, malate, and aspartate represented the maximum level of labelling. All measured metabolites represent the average of three biological replicates +/- standard errors.

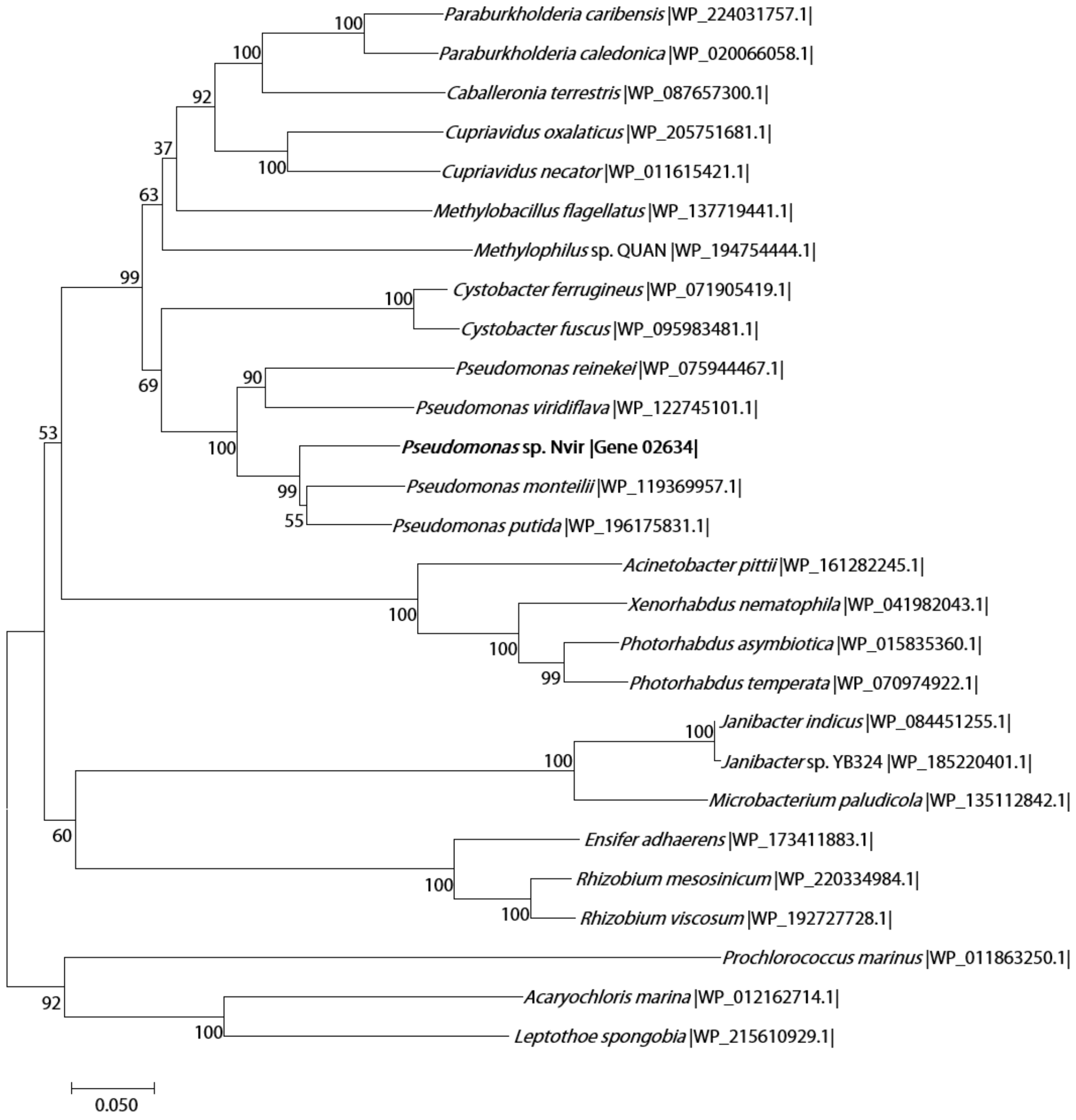

Supplementary Figure 6. Distribution of the *pnm*R gene according to its amino acid sequence. Amino acid sequences were obtained from a BLASTp search from the NCBI database. Sequences were aligned with MUSCLE and a phylogenetic tree was constructed with MEGA7. The evolutionary history was inferred with the Neighbor-Joining method with 500 bootstraps.

Supplementary Materials & Methods

Synthesis of labelled ^{15}N-and 1-^{13}C-nitropropionic acid

All reagents were obtained from commercial suppliers and were used without purification. The ^{1}H-NMR and ^{13}C-NMR were recorded at 298K on a Bruker Avance III 400 (400 MHz) or 500 (500 MHz) spectrometers. The same holds true for Correlation Spectroscopy (COSY), Heteronuclear single-quantum correlation spectroscopy (HSQC) and Heteronuclear multiple-bond correlation spectroscopy (HMBC), which were used for compound assignment. The chemical shifts in the spectra are reported in parts per million (ppm) relative to tetramethylsilane (TMS). The NMR data are presented in the following order: chemical shifts, multiplicity (s = singlet, d = doublet, t = triplet, td = triplet of doublets, m = multiplet and/or multiple resonances), coupling constants J in hertz (Hz), number of protons. The reactions were monitored by TLC-analysis using Silicagel F254 (Merk KGaA) by dipping in $KMnO_4$ followed by charring at ca. 150 °C. Purification by column chromatography was carried out using silica gel 60 (Merck, 0.040- 0.063 mm) or Automatic flash column chromatography on a Biotage Isolera Spektra One using SNAP or Silicycle cartridges (Biotage, 30-100 µm, 60 Å) 4g.

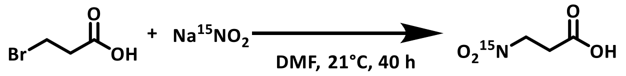

1: 13%

3-(nitro-^{15}N)propanoic acid (1): 3-bromopropionic acid (191.6 mg, 1.25 mmol, 1 equiv.) was added to a stirred suspension of sodium nitrite-^{15}N (104.3 mg, 1.49 mmol, 1.2 equiv.) in dry dimethylformamide (DMF, 5 mL) and the solution was stirred at room temperature for 40 hours. The reaction mixture was diluted with water (10 ml), adjusted to pH 1 with a 1M aqueous HCl solution and extracted with diethyl ether (6x15 ml). The combined organic layers were washed with brine (saturated NaCl aqueous solution; 50 mL), dried over $MgSO_4$ and evaporated *in vacuo*. The crude product was purified using column chromatography (EtOAc:Heptane, 0 to 100%), affording the desired product as a white solid (19 mg, 13%). ^{1}H NMR (500 MHz, MeOD)

δ: 4.72 – 4.64 (m, 2H), 2.98 – 2.92 (m, 2H). ^{13}C NMR (126 MHz, MeOD) δ: 173.45, 71.01 (d, J = 8.2 Hz), 31.71.

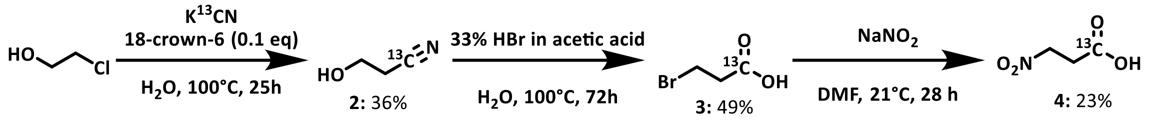

3-hydroxypropanenitrile-1-^{13}C (2): A solution of 2-chloroethanol (0.167 mL, 200 mg, 2.48 mmol, 1 equiv.), potassium cyanide-^{13}C (171 mg, 2.59 mmol, 1.04 equiv.) and 18-crown-6 (65.7 mg, 0.248 mmol, 0.1 equiv.) in water (5 mL) was refluxed for 25h. The solution was cooled to room temperature, extracted with EtOAc (6x10 mL) and dried over $MgSO_4$. The combined organic layers were evaporated and the crude product was purified using column chromatography (EtOAc:Heptane, 0 to 100%), affording the desired product as colourless liquid (64.3 mg, 36%). ^{1}H NMR (400 MHz, CDCl3) δ: 3.88 (q, J = 6.3 Hz, 1H), 2.65 – 2.55 (m, 1H).

3-bromopropanoic-1-^{13}C acid (3): A solution of 3-hydroxypropanenitrile-1-^{13}C (64.3 mg, 0.89 mmol) in a mixture of water (2 mL) and a 33% solution of HBr in acetic acid (3 mL) was heated at reflux. After 26h, another 3 mL of 33% HBr solution in acetic acid was added to the reaction mixture. The reaction was stopped after a total of 72h of reflux, cooled to room temperature, diluted with water (15 mL) and extracted with diethyl ether (6x15 mL). The combined organic layers were dried over $MgSO_4$ and evaporated *in vacuo*. The crude was purified using column chromatography (EtOAc:Heptane, 0 to 100%), affording the desired product (67.2 mg, 49%). ^{1}H NMR (400 MHz, CDCl3) δ: 11.31 (s, 1H), 3.57 (td, J = 6.8, 5.1 Hz, 1H), 3.04 – 2.94 (m, 1H).

3-nitropropanoic-1-^{13}C acid (4): 3-bromopropanoic-1-^{13}C acid (67.2 mg, 0.44 mmol, 1 equiv.) was added to a stirred suspension of sodium nitrite (59.2 mg, 0.96 mmol, 2 equiv.) in dry DMF (5 mL) and the solution was stirred at room temperature for 28 hours. The reaction mixture was diluted with water (10 ml), adjusted to pH 1 with a 1M aqueous HCl solution and extracted with EtOAc (6x15 ml). The combined organic layers were washed with brine (50 mL), dried

over $MgSO_4$ and evaporated in vacuo. The crude product was purified using column chromatography (EtOAc:Heptane, 0 to 100%), affording the desired product (12.4 mg, 23%). ^{1}H NMR (500 MHz, MeOD) δ 4.71 – 4.63 (m, 2H), 2.99 – 2.91 (m, 2H). ^{13}C NMR (126 MHz, MeOD) δ: 173.5, 71.0, 31.7 (d, J = 53.3 Hz).

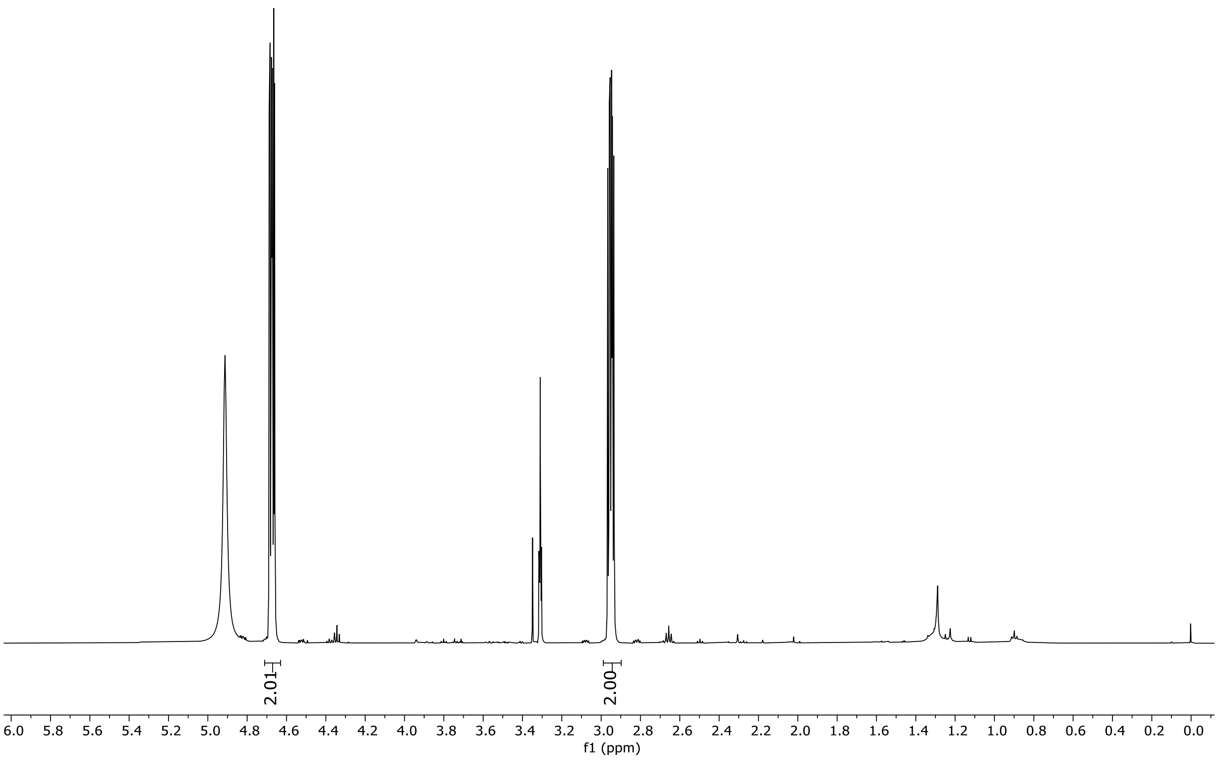

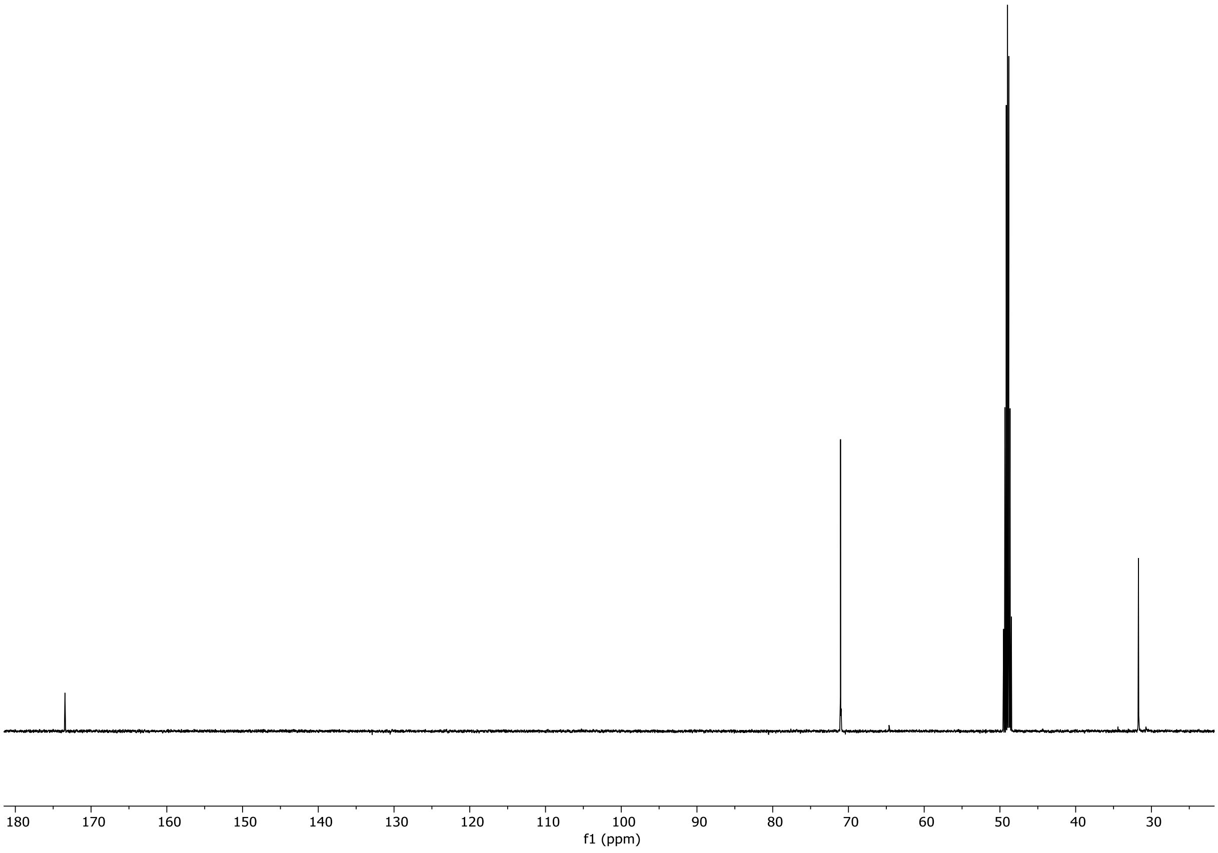

Figure 1: ^{1}H- and ^{13}C-NMR spectra of 3-(nitro-^{15}N)propanoic acid (1)

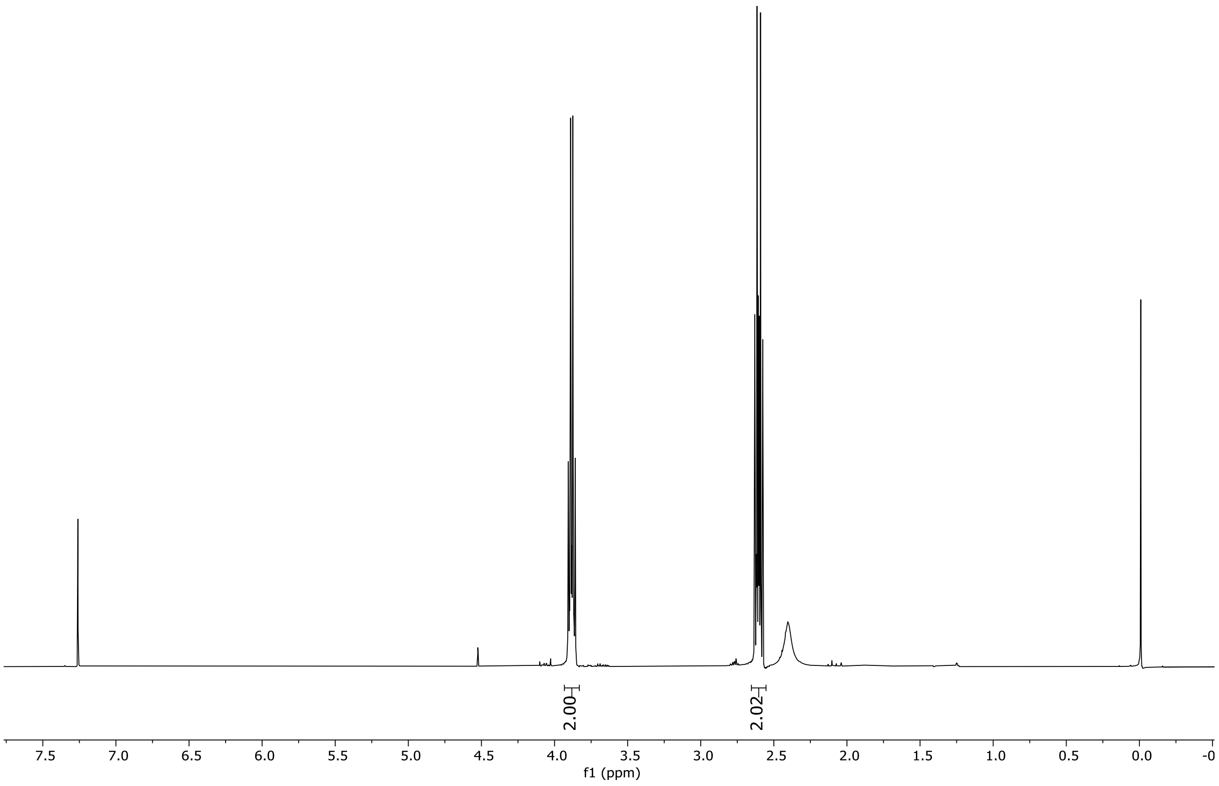

Figure 2: ^{1}H-NMR spectra of 3-hydroxypropanenitrile-1-^{13}C (2)

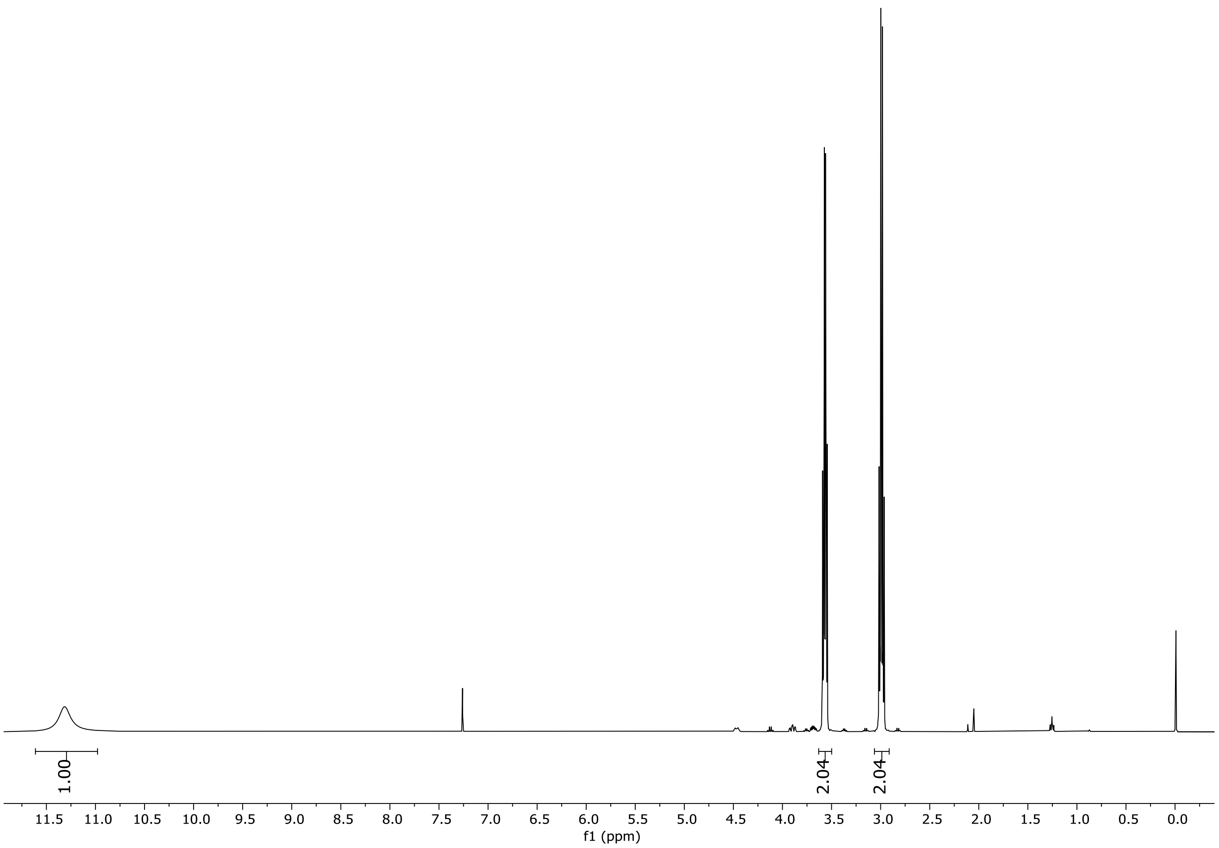

Figure 3: ^{1}H-NMR spectra of 3-bromopropanoic-1-^{13}C acid (3)

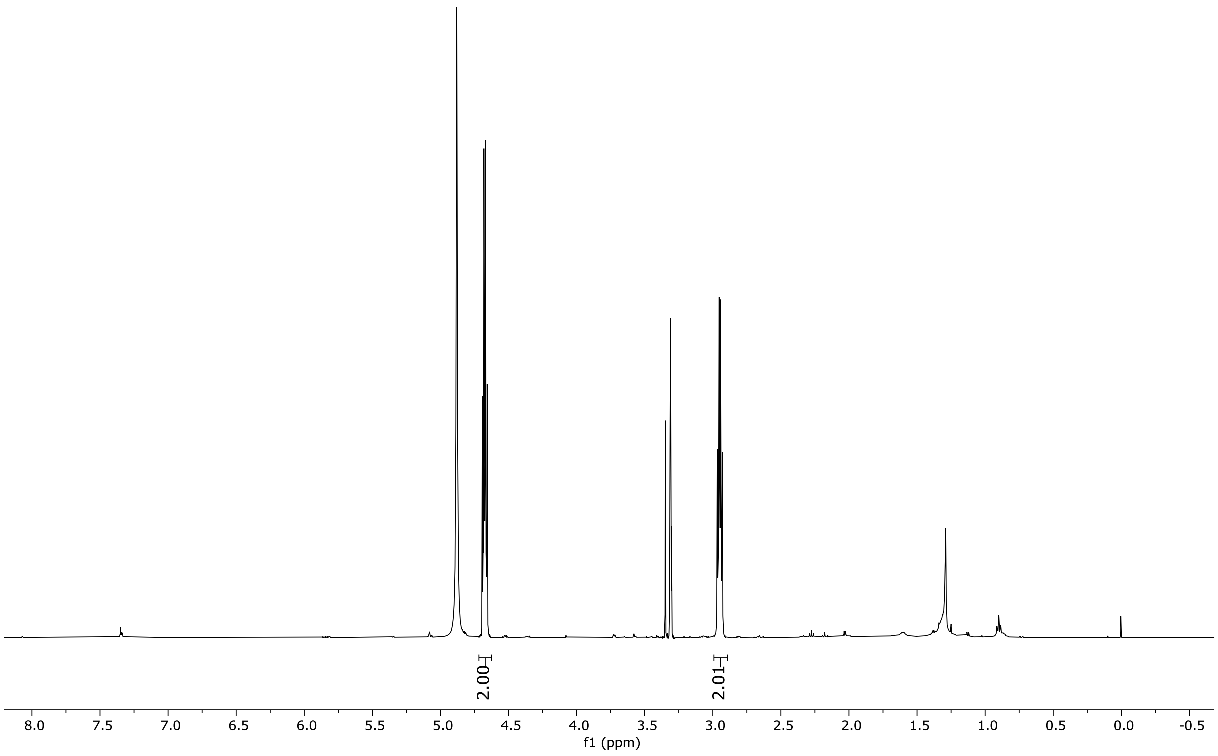

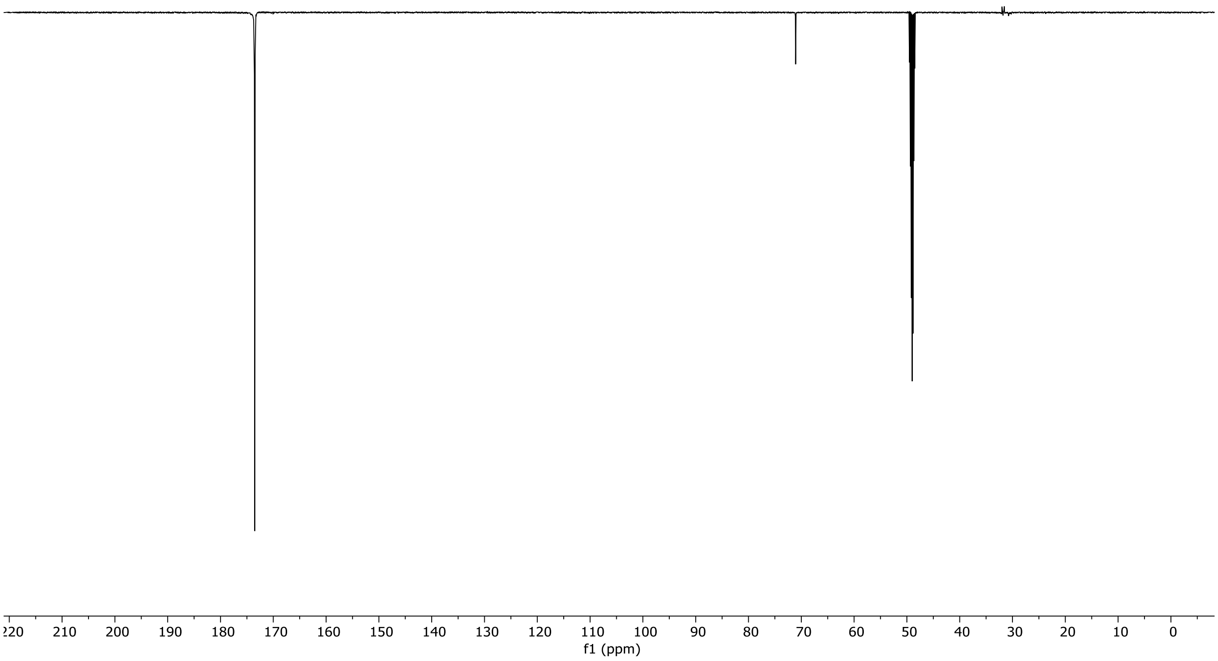

Figure 4: ^{1}H- and ^{13}C-NMR spectra of 3-nitropropanoic-1-^{13}C acid (4)

Supplementary literature

1. Goris, J., K. T. Konstantinidis, J. A. Klappenbach, T. Coenye, P. Vandamme and J. M. Tiedje (2007). "DNA-DNA hybridization values and their relationship to whole-genome sequence similarities." Int J Syst Evol Microbiol **57**(Pt 1): 81-91.
2. Rodriguez-R, L. M. and K. T. Konstantinidis (2016). The enveomics collection: a toolbox for specialized analyses of microbial genomes and metagenomes, PeerJ Preprints.
